# Neurophysiology of Sleep-Deprivation Part 1: Effects of Sleep-Deprivation on Event-Related Potentials (ERPs)—Systematic and Mechanistic Review

**DOI:** 10.3390/jcm15124576

**Published:** 2026-06-12

**Authors:** James Chmiel, Jarosław Nadobnik

**Affiliations:** Faculty of Physical Culture and Health, Institute of Physical Culture Sciences, University of Szczecin, Al. Piastów 40B blok 6, 71-065 Szczecin, Poland; jaroslaw.nadobnik@usz.edu.pl

**Keywords:** sleep deprivation, sleep, event-related potentials, EEG, electroencephalography, electroencephalogram, neurophysiology, circadian rhythm

## Abstract

**Background:** Sleep deprivation is one of the major public health and performance risk factors, with documented effects on vigilance, executive function, emotional regulation, and safety-critical behaviour. This review examines how event-related potentials (ERPs)—which provide millisecond-level resolution of cognitive processing stages—can clarify which neural processes are most affected by sleep loss, from early sensory encoding to later evaluative and control-related stages. **Materials and Methods:** This study was conducted as a systematic review of human studies on sleep deprivation and ERPs. Eligible studies included human participants, focused primarily on acute/total sleep deprivation, and reported ERP outcomes (e.g., amplitude, latency, topography, or related event-locked EEG measures). Searches were performed in major biomedical/psychology databases using sleep deprivation and ERP terms, with additional forward/backward citation searching. Data was extracted in a structured format (participant characteristics, deprivation protocol, ERP methods, behavioural outcomes, ERP findings, and recovery/countermeasure effects). Due to substantial heterogeneity in paradigms, protocols, and ERP methods, findings were synthesised narratively rather than meta-analysed. Risk of bias was assessed with RoB 2 and ROBINS-I. **Results:** The search identified 854 records, of which 82 studies were included following deduplication, screening, full-text review, and citation chasing. Samples were typically small, highly selected, and dominated by healthy young adults, with frequent attrition related to prolonged wakefulness and EEG data-quality constraints. Across studies, sleep deprivation produced stage-specific and task-dependent ERP effects rather than a single uniform pattern. The most consistent findings involved mid-to-late components. These components typically showed prolonged latency and reduced amplitude. In some cases, amplitude increases were observed and interpreted as compensatory recruitment. Early sensory/pre-attentive components (e.g., P1/N1/MMN/P50) were often relatively preserved, but showed selective vulnerability, including latency slowing, reduced filtering/gating, or decreased phase locking. A recurring observation was a behaviour–ERP dissociation, where ERP abnormalities were detectable even when behavioural impairment was modest, indicating covert neural inefficiency or compensation. Recovery sleep, naps, and countermeasures (e.g., modafinil, caffeine) produced partial, component-specific recovery, with amplitude and latency often recovering at different rates. **Conclusions:** The evidence indicates that sleep deprivation primarily disrupts higher-order, late-stage, and temporally coordinated neural processing, while earlier sensory processing is often preserved but becomes slower and less stable. Among ERP markers, the P300/P3 family is the most robust and informative signature of sleep loss effects and recovery. ERPs are therefore a sensitive tool for detecting neural dysfunction and compensation under sleep deprivation, including changes that may precede overt behavioural decline. Future research must improve the generalisability and reproducibility of ERP findings by employing larger, more diverse samples, alongside more standardised methodological, recording, and reporting practices.

## 1. Introduction

Sleep deprivation refers to an insufficient quantity and/or quality of sleep that fails to meet an individual’s physiological needs for optimal daytime functioning. In experimental and clinical literature, sleep loss is commonly operationalised as acute total sleep deprivation (continuous wakefulness for one or more nights) or chronic partial sleep restriction (repeated nights with curtailed sleep opportunity), with the latter often considered a closer analogue of real-world patterns in modern societies [1,2]. More broadly, ‘sleep deprivation’ encompasses heterogeneous conditions, including voluntary sleep curtailment, occupational demands, and environmental disruption. These conditions vary in timing, chronicity, and degree of circadian misalignment. Each of these factors shapes behavioural and biological outcomes [2,3].

A convergent finding across laboratory sleep-loss paradigms is that reduced sleep produces state instability: wakefulness becomes harder to sustain, leading to moment-to-moment fluctuations in alertness, attentional lapses, and “microsleeps” [3,4]. These deficits are especially prominent in tasks requiring sustained attention and rapid, consistent response, and they generalise from acute deprivation to chronic restriction in a dose dependent manner [5,6,7]. A key feature of chronic sleep restriction is that performance impairments can accumulate across days, while subjective sleepiness may plateau or underestimate objective decline, increasing risk in safety-critical contexts [1,7]. Reviews of neurocognitive consequences emphasise robust effects on vigilance, processing speed, working memory, and aspects of executive control, with inter-individual variability influenced by trait-like vulnerability and contextual factors [3,8,9].

Sleep loss has well-documented implications for health and public safety. In applied comparisons, extended wakefulness can yield performance degradation comparable to legally relevant levels of alcohol intoxication, underscoring the societal importance of sleep-related fatigue management in transportation and occupational settings [10,11]. Beyond performance, insufficient sleep is increasingly linked to adverse cardiometabolic outcomes. Controlled sleep debt experiments demonstrate alterations in metabolic and endocrine function, including changes in carbohydrate metabolism and hormonal regulation [12,13]. Observational work similarly associates short habitual sleep with appetite-related hormonal profiles (e.g., leptin/ghrelin) that may promote increased energy intake, and with higher body mass index [12,14]. Integrative reviews further connect sleep curtailment to insulin resistance, dyslipidaemia, and related metabolic risk pathways [15,16]. At the population level, systematic reviews and meta-analyses report associations between sleep duration and mortality and cardiometabolic outcomes, including cardiovascular disease risk markers and endpoints [17,18].

Sleep deprivation also interacts bidirectionally with the immune and inflammatory systems. Contemporary reviews describe how sleep modulates host defence via neuroendocrine and autonomic pathways, and how insufficient or disturbed sleep can contribute to low-grade inflammation implicated in chronic disease vulnerability [19,20]. Quantitative syntheses show links between sleep disturbance (and extremes of sleep duration) and inflammatory markers across cohorts and clinical samples [21]. These mechanistic connections provide a plausible biological substrate through which persistent sleep loss may influence long-term morbidity beyond its immediate neurobehavioural manifestations [15,22].

Affective functioning is similarly sensitive to sleep loss. Conceptual models and empirical reviews argue that sleep supports emotional brain regulation, and that insufficient sleep alters emotional reactivity, appraisal, and regulation, with implications for mood and anxiety symptomatology [23,24,25]. Importantly, sleep need varies by age and individual factors; consensus recommendations for healthy adults emphasise that obtaining sufficient sleep is foundational for optimal health and functioning, providing a normative framework against which “sleep deprivation” is often defined in applied research [26].

Event-related potentials (ERPs) are voltage fluctuations derived from the electroencephalogram (EEG) that are time-locked to internal or external events, such a stimulus onset, decision points, or motor responses. ERPs are typically obtained by segmenting continuous EEG into epochs around events of interest and averaging across trials (or applying related estimation approaches) to enhance event-related signal components relative to ongoing activity and noise [27,28]. As ERPs provide millisecond-scale temporal resolution, they are uniquely suited to tracking the timing of perceptual encoding, attentional selection, response preparation, and post-response evaluation—processes that can be difficult to resolve with slower hemodynamic measures [27,29].

**Figure 1:**
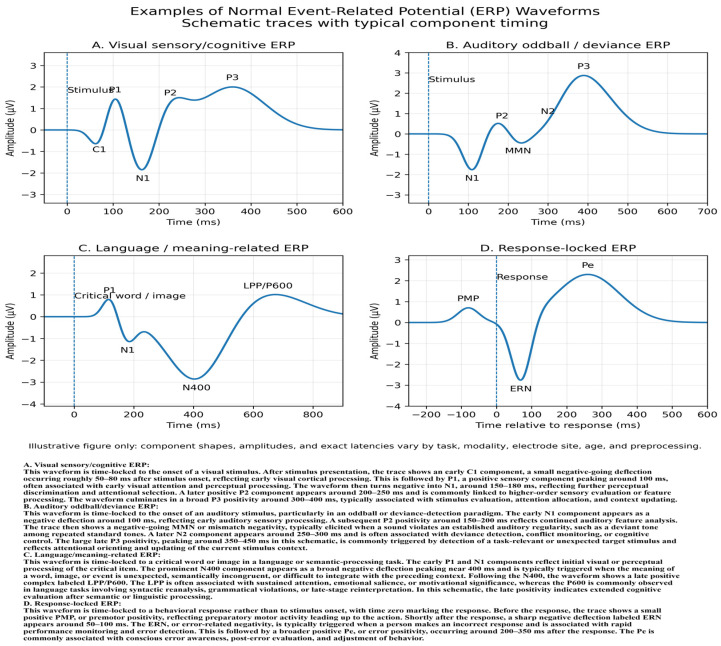
Examples of normal event-related potential waveforms.

ERP waveforms are conventionally described in terms of components—deflections characterised by polarity (positive/negative), latency, scalp distribution, and sensitivity to experimental manipulations. Well-studied examples include early sensory components (often reflecting initial perceptual processing) and later components associated with attention and context updating [27,30]. Among late components, the P300 (including P3a and P3b subcomponents) has been extensively linked to attentional allocation and memory-related updating operations, and its amplitude/latency characteristics have been interpreted within integrative neurocognitive models [30]. Interpreting ERP components requires careful control of multiple methodological factors. These include experimental design, referencing, filtering, baseline correction, artifact handling, and measurement choices [28,31]. Figure 1 shows examples of normal event-related potential waveforms and schematic traces with typical component timing.

A persistent challenge in ERP research is the presence of artifacts from eye blinks, saccades, muscle activity, and environmental noise. Foundational correction approaches include regression-based procedures for offline ocular artifact removal, enabling retention of trials that would otherwise be discarded [32]. In parallel, modern preprocessing toolchains often incorporate independent component analysis and related decomposition techniques; widely used open-source software such as EEGLAB has supported standardised, transparent workflows for single-trial and averaged EEG/ERP analysis [33]. These advances have improved data quality and reproducibility, but they also increase the importance of explicitly reporting analytical decisions, given that preprocessing choices can influence component morphology and measurement [28,31].

Sleep deprivation perturbs neurobehavioural and physiological systems that map naturally onto ERP-relevant constructs: vigilance and attentional stability, speeded information processing, executive control, affective appraisal, and performance monitoring. ERPs offer a mechanistically informative lens on such functions by decomposing behaviour into temporally specific processing stages, potentially revealing whether sleep loss primarily alters early sensory encoding, attentional selection, evidence accumulation, response preparation, or post-response evaluative processes. At the same time, because sleep deprivation affects broad neurophysiological states (e.g., arousal, homeostatic sleep pressure, and neuromodulatory tone), rigorous ERP interpretation requires careful control of confounds and adherence to methodological standards.

## 2. Materials and Methods

This review was conducted as a systematic review of human studies examining the effects of sleep deprivation on ERPs. The review question was structured to identify experimental and observational studies in which acute sleep deprivation was the exposure and ERP-derived indices (e.g., amplitude, latency, topography, or related event-locked EEG measures) were the primary or secondary outcomes. The workflow included database searches, deduplication, title/abstract screening, full-text eligibility assessment, manual citation screening, structured data extraction, and risk-of-bias appraisal. The final review corpus consisted of studies published from 1980 onward, with a strong emphasis on laboratory total sleep deprivation paradigms and ERP-based cognitive or affective task designs. The PRISMA checklist is presented in Supplementary Table S1.

### 2.1. Search Strategy and Information Sources

A systematic literature search was performed in major biomedical and psychology databases (PubMed/MEDLINE, Scopus, Web of Science, PsycINFO, ResearchGate and Google Scholar), using combinations of controlled vocabulary and free-text terms related to sleep deprivation and ERPs. We searched for studies published from 1 January 1980 to 1 January 2026. Core search concepts included the following:

- sleep deprivation/total sleep deprivation/sleep loss/prolonged wakefulness
- event-related potentials/ERP/EEG evoked potentials/P300/N2/CNV/MMN/ERN/Pe

The search strategy was supplemented by the following:

1. Reference list screening of eligible articles, and
2. Forward and backward citation searching (“similar,” cited, and citing articles), which yielded additional studies beyond the database search. This supplementary step is explicitly reflected in the review flow description.

All records were imported into EndNote for reference management and deduplication.

The database search strategy was developed to capture studies examining the effects of sleep deprivation on ERPs in humans. Searches combined controlled vocabulary terms (where available, e.g., MeSH in PubMed) with free-text keywords and synonyms, using Boolean operators (AND, OR) and truncation/wildcards adapted to each database interface. The two core concept blocks were: (1) sleep deprivation/sleep loss and (2) ERP/event-related potentials. This approach expands the current draft list of core concepts (sleep deprivation/total sleep deprivation/sleep loss/prolonged wakefulness; ERP/EEG evoked potentials/P300/N2/CNV/MMN/ERN/Pe) into a reproducible search syntax.

A representative search structure was as follows:

Sleep deprivation terms:

(“sleep deprivation” OR “total sleep deprivation” OR “sleep loss” OR “prolonged wakefulness” OR “sleep-disrupted” OR “sleep restriction”)

ERP terms:

(“event-related potential*” OR ERP OR “evoked potential*” OR “EEG evoked potential*” OR P300 OR P3 OR N1 OR P1 OR N2 OR P2 OR MMN OR “mismatch negativity” OR CNV OR “contingent negative variation” OR ERN OR Ne OR Pe OR LPP)

Combined strategy:

(Sleep deprivation terms) AND (ERP terms)

Search strings were tailored to the syntax and indexing system of each database, while preserving the same conceptual structure across sources.

### 2.2. Eligibility Criteria

Studies were selected according to predefined inclusion and exclusion criteria.

#### 2.2.1. Inclusion Criteria

Studies were eligible if they

- included human participants (healthy or clinical populations),
- examined sleep deprivation (primarily acute/total sleep deprivation),
- reported ERP outcomes derived from EEG (including standard ERP components such as P1/N1, P2/N2, P3/P300, CNV, MMN, ERN/Ne, Pe, LPP, etc.),
- used an experimental, quasi-experimental, or observational design with analysable ERP data,
- were published in English, and
- were available as full-text publications.

#### 2.2.2. Exclusion Criteria

Studies were excluded if they

- examined partial sleep deprivation rather than the target exposure (total sleep deprivation),
- focused on sleep fragmentation instead of sleep deprivation,
- were animal studies (e.g., rat experiments),
- were non-English publications,
- were posters, study protocols, or reviews,
- or were published before 1980.

These exclusion categories are directly consistent with the reasons reported in the screening results.

### 2.3. Study Selection Process

Study selection was performed in two stages:

1. Title and abstract screening, followed by
2. Full-text review of potentially eligible records.

### 2.4. Data Extraction

A structured data-extraction approach was used to summarise the included studies (presented in Table 1). For each study, the following information was extracted where available:

- Number of study
- Study design (within-subject, between-group, crossover, intervention/countermeasure design)
- Participant characteristics (sample size, age, sex, population type, inclusion/exclusion criteria)
- Sleep deprivation protocol (duration of wakefulness, recovery sleep, naps, pharmacological countermeasures, control conditions)
- Task/paradigm (oddball, Go/NoGo, stop-signal, flanker, N-back, emotional tasks, vigilance tasks, etc.)
- ERP methodology (components analysed, scalp sites/regions, amplitude/latency windows, preprocessing/artifact handling when reported)
- Behavioural outcomes (accuracy, reaction time, errors, vigilance metrics)
- Main ERP findings (direction of effects, latency/amplitude changes, topographic or condition-specific differences)
- Recovery/countermeasure effects (if applicable)

### 2.5. Data Synthesis

Given the substantial heterogeneity across studies in

- sleep deprivation duration and protocols,
- participant populations (healthy, clinical, specialised occupational/athlete cohorts),
- ERP tasks and cognitive domains,
- ERP preprocessing and measurement conventions,
- and outcomes reported (different components, latency vs. amplitude, stimulus-locked vs. response-locked analyses),

The findings were synthesised using a narrative/descriptive approach rather than meta-analysis. Studies were grouped and interpreted by:

1. Participant/sample characteristics,
2. ERP paradigm/task family, and
3. ERP component domain (e.g., P3/P300, early sensory/pre-attentive components, P2/N2, ERN/Pe, CNV, affective and memory-related components).

### 2.6. Risk of Bias Assessment

Risk of bias was assessed separately for randomised and non-randomised studies using standard tools:

- RoB 2 for randomised studies, and
- ROBINS-I for non-randomised studies.

### 2.7. Registration of a Systematic Review

This review has been registered in the PROSPERO database (CRD420261340666).

## 3. Results

**Figure 2:**
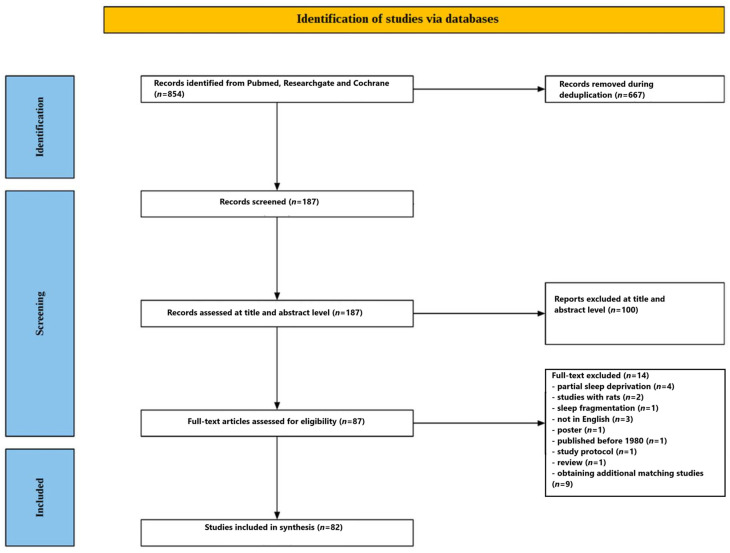
Flow chart depicting the different phases of the systematic review.

Figure 2 provides a summary of the screening process. The literature search identified 854 articles. Deduplication was performed using EndNote, which resulted in the removal of 667 duplicates. 187 articles were assessed based on title and abstract reading. At this stage, 100 articles were excluded because they did not examine the effect of sleep deprivation on ERPs. Eighty-seven articles were advanced to full-text review. At this stage, 14 articles were excluded. Four studies were excluded because they examined partial sleep deprivation, two studies because they studied rats, one study because it examined sleep fragmentation, three studies because they were written in a language other than English, one study because it was a poster, one study because it was published before 1980, one study because it was a study protocol, and one study because it was a review. Seventy-three articles were found that fit the scope of the review. A search for similar, cited, and citing articles yielded an additional 9 articles. Ultimately, 82 articles were included in the review [34,35,36,37,38,39,40,41,42,43,44,45,46,47,48,49,50,51,52,53,54,55,56,57,58,59,60,61,62,63,64,65,66,67,68,69,70,71,72,73,74,75,76,77,78,79,80,81,82,83,84,85,86,87,88,89,90,91,92,93,94,95,96,97,98,99,100,101,102,103,104,105,106,107,108,109,110,111,112,113,114,115]. The included studies are presented in Table 1.

### 3.1. Participants’ Characteristics

Across the reviewed ERP sleep deprivation studies ([34,35,36,37,38,39,40,41,42,43,44,45,46,47,48,49,50,51,52,53,54,55,56,57,58,59,60,61,62,63,64,65,66,67,68,69,70,71,72,73,74,75,76,77,78,79,80,81,82,83,84,85,86,87,88,89,90,91,92,93,94,95,96,97,98,99,100,101,102,103,104,105,106,107,108,109,110,111,112,113,114,115]), participant samples were predominantly healthy young adults, with a strong concentration of university students and a frequent overrepresentation of men, especially in tightly controlled laboratory studies of total sleep deprivation (TSD). This pattern was evident in both early psychophysiological work and more recent ERP experiments. Many studies recruited participants in their late teens to late twenties and selected relatively homogeneous samples to reduce inter-individual variability in sleep habits, cognitive performance, and ERP morphology. In several cases, samples were restricted to young men only (e.g., [34,35,44,46,47,48,49,52,53,54,56,57,62,63,64,66,69,71,78,79,85,91,92,94,96,98,100,102,103,105,106,107,112,113,115]), often with additional constraints such as right-handedness, good sleep quality, and regular sleep schedules. These choices enhanced experimental control and improved ERP signal consistency. However, they limited generalisability to women, older adults, and more heterogeneous or clinical populations. Despite this dominant pattern, participant profiles were not uniform. A substantial subset of studies used mixed-sex young adult samples (e.g., [37,38,39,40,41,58,61,65,67,68,72,73,75,77,80,81,82,87,88,90,93,95,97,101,104,108,109,110,111]), and a smaller number recruited female-only samples (e.g., [70,83,86]). Even in mixed-sex studies, however, sample sizes were often modest and not powered for sex-specific analyses. This uneven sex distribution is an important characteristic of the literature because sex and hormonal status may influence sleep-loss vulnerability, affective processing, pain responses, and ERP components. Only a few studies explicitly handled these factors in detail (e.g., menstrual cycle control in pain-evoked ERP work [104]). Overall, the field has favoured homogeneity over representativeness, although more recent studies increasingly include both sexes.

Age distributions were similarly concentrated. Most studies recruited participants aged approximately 18–30 years, with mean ages typically in the early 20s. This was especially common in student-based paradigms examining working memory, executive control, selective attention, and emotional processing. Nonetheless, several studies broadened the age range or explicitly examined age effects. For example, professional truck drivers in [36] ranged from 23 to 62 years (mean 45.3), novice and older licensed drivers were compared in [50], and [81] directly contrasted younger (20–30 years) and older (60–70 years) adults. Clinical or occupational samples also increased age heterogeneity, such as physicians with nightshift duties in [64], depressive inpatients in [83] (23–70 years), and narcoleptic patients plus middle-aged controls in [109]. These studies are especially valuable because they improve ecological and clinical relevance, but they also introduce participant-level factors (age, occupation, diagnosis, medication status) that complicate direct comparison with standard young-student samples.

A defining characteristic across the literature is the use of strict inclusion and exclusion criteria. Most studies recruited participants free from neurological disease, psychiatric illness, diagnosed sleep disorders, major medical conditions, and recent use of medications or substances that could affect sleep or cognition. Common exclusions included psychoactive medication use, substance abuse, excessive daytime sleepiness, irregular sleep schedules, shift work, and extreme chronotypes. Many studies also required normal or corrected-to-normal vision for visual paradigms and normal hearing for auditory paradigms; some auditory studies used detailed audiological screening (e.g., otoscopy, tympanometry, audiogram in [38]) or polysomnographic confirmation of baseline sleep health (e.g., [42]). These modality-specific controls strengthened internal validity by reducing the likelihood that ERP differences reflected sensory deficits rather than sleep deprivation.

Many investigations further selected for high-functioning, highly screened participants, particularly in studies focused on executive function or working memory. In addition to general health, investigators often required good habitual sleep quality (commonly via PSQI cutoffs such as <5 or <7), stable sleep hygiene, and regular bed/wake times. Some studies also screened for chronotype, trait anxiety, depression symptoms, daytime sleepiness, or cognitive ability using measures such as the Morningness–Eveningness Questionnaire, BDI, ESS, HAMD/HAMA, and Raven’s matrices ([64,67,68,69,70,71,73,76,82,86,88,93,100,103,105]). In several cases, unusually stringent criteria were used, including high IQ and memory thresholds [67,71,105] or exclusion of strong morning/evening types and habitual nappers [34,63]. These procedures produced methodologically “clean” samples and likely reduced noise in ERP data, but they may also have selected for individuals who are more resilient or less representative of the general population.

Handedness was controlled in many studies, especially those emphasising ERP topography, hemispheric asymmetry, or executive-function processes. A large proportion explicitly required right-handed participants (e.g., [35,37,39,42,44,45,46,47,48,49,52,56,57,58,59,61,64,65,66,68,69,71,72,75,77,82,83,91,94]). This likely reflects a desire to reduce variance associated with lateralised cortical organisation, particularly in source localisation or frontal-control paradigms. While scientifically defensible, it further narrows participant representativeness.

Another recurring feature was the normalisation of habitual sleep before deprivation. Many studies required 1–2 weeks of regular sleep prior to testing, often verified with sleep diaries/logs and sometimes actigraphy, polysomnography, or adaptation nights in the laboratory [34,46,55,62,94,95,98,100,103,104,108,111,112]. This was frequently paired with strict abstinence requirements for caffeine, alcohol, nicotine, medications, and naps before and during the study [34,37,39,46,48,52,57,62,63,93,96,98,100,102,103,111,112,115]. A few studies allowed carefully controlled caffeine to avoid withdrawal effects [45,105]. Such run-in controls are important because ERP components (e.g., CNV, N2, P3, ERN, N400) are sensitive to baseline fatigue, circadian misalignment, and chronic sleep restriction, but these controls again bias samples toward participants with stable, compliant lifestyles.

Although most studies excluded shift workers and chronically sleep-restricted individuals, a few deliberately incorporated sleep history or occupational sleep exposure as variables of interest. Ref. [42] compared participants with and without long-term nightshift history to assess whether chronic sleep disruption altered vulnerability to acute TSD, and ref. [64] recruited physicians with regular nightshift duties, directly targeting occupational fatigue. Similarly, ref. [45] stratified participants by baseline sleep quality (good vs. poor sleepers) within an otherwise healthy sample. These designs are notable because they acknowledge trait-level and lifestyle-related moderators of sleep-loss effects rather than treating all “healthy” participants as equivalent.

The literature also includes several specialised populations that extend beyond standard student cohorts. These include professional truck drivers ([36]), military cadets and soldiers ([43]), younger and older licensed drivers ([50]), novice drivers ([77]), table tennis athletes and non-athlete controls [56,115], winter-sport athletes ([108]), physicians ([64]), depressive inpatients ([83]), and narcoleptic patients with controls ([109]). Such studies improve ecological validity by situating ERP responses in contexts like driving safety, military readiness, athletic performance, and clinical sleep pathology. However, they also introduce population-specific characteristics (such as training, occupational demands, diagnosis, medication use, age, and motivation) that may influence both vulnerability and compensatory responses to sleep loss.

Sample sizes across the corpus were generally small to moderate, which is typical for ERP sleep deprivation research given the intensive nature of overnight monitoring, repeated testing, and EEG preprocessing. Many studies analysed roughly 10–20 participants after exclusions (e.g., [34,38,46,47,48,49,53,55,59,60,62,63,65,68,70,72,74,75,79,85,90,92,94,97,104,114]). Some studies were especially small (e.g., *n* = 5 in [92], *n* = 8 in [66]), while larger cohorts appeared in [37,40,44,50,51,57,71,77,78,82,93,95,103], and [108]. Even in larger studies, subgrouping by condition, age, incentives, athlete status, or clinical response often reduced the effective sample size for key comparisons.

A related and important participant characteristic was attrition and post hoc data exclusion. Many studies reported losses due to EEG artefacts, excessive blink/movement contamination, insufficient valid trials, technical failures, noncompliance, poor behavioural performance, dropout, or inability to sleep in the laboratory. This pattern was common across older and newer work (e.g., [38,41,46,48,49,53,56,59,60,65,67,71,73,77,79,80,82,87,91,93,96,98,113]). As drowsiness and fatigue increase movement, microsleeps, and ocular artefacts, final ERP-analysed samples were often meaningfully smaller than initially recruited cohorts. This has direct implications for statistical power, especially in subgroup analyses and interaction tests.

Another participant-related feature across many studies was strict monitoring during deprivation, which functionally characterises the sample at the time of ERP testing. TSD and partial sleep deprivation protocols typically involved continuous supervision by research staff, restrictions to low-arousal activities, and repeated checks to prevent napping. Many studies also repeatedly measured subjective sleepiness (e.g., SSS, KSS, VAS) and sometimes included physician oversight or frequent experimenter monitoring [67,69,91]. This repeated state monitoring is relevant because it documents the participants’ actual sleepiness and impairment level at ERP acquisition, rather than assuming a uniform response to the same nominal sleep-loss duration.

### 3.2. ERP Paradigms

Across the reviewed studies ([34,35,36,37,38,39,40,41,42,43,44,45,46,47,48,49,50,51,52,53,54,55,56,57,58,59,60,61,62,63,64,65,66,67,68,69,70,71,72,73,74,75,76,77,78,79,80,81,82,83,84,85,86,87,88,89,90,91,92,93,94,95,96,97,98,99,100,101,102,103,104,105,106,107,108,109,110,111,112,113,114,115]), ERP paradigms varied widely in their implementation. Despite this variability, they shared a common goal: isolating specific stages of information processing. They also tested whether sleep deprivation selectively alters these stages. These stages ranged from early sensory encoding to higher-order operations such as attentional orienting, conflict monitoring, inhibitory control, error detection, affective evaluation, semantic integration, and motor preparation. In practice, the literature converged on several recurring paradigm families: oddball/target-detection tasks, vigilance and sustained-attention paradigms, Go/NoGo and other inhibitory-control tasks, conflict/task-switching and executive-control paradigms, performance-monitoring/error paradigms, working-memory updating tasks (especially N-back), CNV warning–imperative paradigms, and a smaller but important set of emotion, empathy, pain, semantic-memory, and applied real-world tasks. This breadth is a major strength of the field because it demonstrates multi-domain ERP sensitivity to sleep loss, although it also complicates direct comparison across studies due to differences in modality, task demands, and component definitions.

A foundational and very common paradigm family was the oddball task, especially in auditory form. Across studies [34,37,38,39,40,41,42,58,61,62,63,66,78,79,85,91], and also in the NeuroCatch platform in [40], participants detected infrequent target tones among frequent standard tones. In some variants, targets were counted silently rather than responded to overtly. These paradigms were primarily used to elicit the P300/P3b as an index of attentional resource allocation and stimulus evaluation, often alongside earlier components such as N1, P2, and N2. Core design features were generally similar (frequent standards, rare targets, fixed or semi-fixed interstimulus intervals, controlled tone intensity/duration), but there were important variations in implementation and interpretation. Some studies used mental counting only [34,37,39,63], whereas others required speeded button presses [38,42], and some expanded the oddball logic to include additional deviant classes. For example, [58] used a novelty oddball (standards, targets, and novel environmental sounds) to dissociate target P3 from the frontocentral novelty P3/P3a, while [40] embedded oddball-derived N100/P300 assessment within a brief clinical EEG protocol that also included semantic processing (N400). Other auditory paradigms extended beyond simple oddball detection, such as [80] (auditory selective attention with attended vs. unattended channels), [81] (dual-stream distraction/reorienting with novel sounds), and [99] (combined P300/MMN with brainstem auditory evoked potentials), showing that auditory ERP designs in this literature span both attention-dependent and pre-attentive processing.

A closely related cluster involved visual oddball, visual discrimination, and target-detection paradigms, used to probe attention and perceptual processing under prolonged wakefulness (e.g., [65,68,74,90]). These tasks followed the same event-related logic as auditory oddball paradigms (rare targets embedded among more frequent events) but used visual targets, often with repeated measurements across sleep deprivation and recovery. They commonly elicited early visual components (e.g., P1/N1) and later attention/evaluation components (especially P3), enabling the authors to test whether sleep loss primarily degrades perceptual encoding, attentional allocation, or both. Studies such as [74] were especially particularly valuable, as they tracked ERP changes over multiple time points (prolonged wakefulness and recovery). This allowed temporal characterisation of both degradation and rebound, beyond a simple deprived-versus-rested comparison.

Another major paradigm family comprised vigilance and sustained-attention tasks, especially the Psychomotor Vigilance Task (PVT) and closely related continuous-attention paradigms ([69,70,76,88,89], and in broader behavioural-ERP contexts [90]). These paradigms are central in sleep deprivation research because they capture the hallmark behavioural phenotype of sleep loss: slowed and unstable responding, lapses, and time-on-task decline. ERP analyses in these tasks typically focused on early sensory components and P300-like responses to target onset, sometimes contrasted across fast trials, slow trials, and lapses. In this domain, the ERP paradigm is less about complex executive operations and more about state instability and fluctuating alertness. Studies such as [69] and [91] also explicitly manipulated time-on-task and circadian timing, which is important because vigilance-related ERP changes can reflect an interaction between sleep pressure, circadian phase, and fatigue accumulation within a task block.

A particularly prominent set of paradigms targeted executive control, inhibition, and conflict processing, often through Go/NoGo or related response-control tasks. Across studies [35,52,53,54,63,64,75,84,86,112], and [113], participants made rapid responses to frequent Go stimuli and withheld responses to NoGo stimuli, enabling extraction of NoGo-N2 and NoGo-P3 as markers of conflict detection and inhibitory control. Although the stimulus materials varied widely (tones, arrows, digits, abstract symbols, triangles), the cognitive structure was consistent: repeated Go trials create a prepotent response tendency, and NoGo trials probe the ability to suppress that tendency under sleep loss. Several studies made important design choices to address interpretive confounds. For example, [54] used a 50/50 Go/NoGo probability to reduce the prepotent-response bias, and [55] (a related inhibition framework) used complementary Go and NoGo blocks so that rare-event probability was equated, helping to distinguish true inhibitory deficits from generic stimulus rarity effects. These distinctions matter because a P3 reduction in a standard oddball task reflects different cognitive operations than a NoGo-P3 change in an inhibition task.

Within the broader executive-control category, a number of studies used conflict and interference paradigms (e.g., flanker-like, Simon, Stroop, emotional conflict) to test whether sleep deprivation impairs top-down control beyond vigilance. Studies [51,72,73,82,97], and [108] are especially relevant here. These paradigms evoke frontocentral N2, P3, and sometimes later positive or negative slow components linked to conflict resolution and control recruitment. Study [73] is notable for adding a motivational manipulation (incentives vs. no incentives), allowing the authors to test whether reward context buffers sleep-loss-related ERP deficits in conflict processing. Study [72] used an emotional conflict structure, blending executive control with affective processing and making it possible to ask whether sleep deprivation disproportionately disrupts control over emotionally salient interference. Study [97], using a Simon conflict/action-monitoring design with EMG and response-locked ERPs, extended this approach by separating proactive control from reactive error monitoring at a finer temporal level.

Closely related are task-switching and cognitive-flexibility paradigms, which were used in [84] and especially [93]. These tasks require participants to alternate between task rules or stimulus-response mappings, enabling decomposition of executive control into preparatory and target-evoked processes. Study [93] used a cue-based switch design (e.g., cue indicating classify by colour vs. shape) and analysed a broad sequence of components: N1/P2 (early attentional and stimulus-response retrieval processes), N2 (conflict/inhibition), P3 (resource allocation/updating), and a late negative component (LNC) linked to sustained maintenance. This multi-component strategy is a good example of how ERP paradigms in the sleep deprivation literature are often designed to test where in the processing stream impairment emerges, rather than merely whether overall performance declines.

Another core paradigm family consisted of working memory updating tasks, especially visual or verbal 2-back paradigms, which were heavily used in [44,46,47,48,49,57,98,100,103,105,115]. These paradigms are especially valuable because they move beyond simple vigilance and require continuous executive updating, maintenance, comparison, and response selection. Most used repeated visual presentations (letters, objects, spatial locations, or sport-relevant spatial stimuli) with standardised timing and match/mismatch decisions, often after substantial pretraining to stabilise baseline performance. ERP analyses in this family focused on a P2/N2/P3 complex (or related early–late sequences), with somewhat different emphases across studies:

- P2 was often interpreted as early selection or perceptual-attentional engagement,
- N2 as comparison/conflict monitoring or control, and
- P3 as updating/resource allocation/context revision.

Some studies dissected subprocesses further by comparing match vs. mismatch trials [44,100,103,105], spatial vs. object updating [47,48], or lower vs. higher load (1-back vs. 2-back in [49]). Others emphasised earlier components (e.g., frontal N1/P2 in [98]) to test whether sleep deprivation and recovery first alter early sensory-attentional stages before later executive updating. Study [115] adapted the 2-back design to a sport-relevant spatial stimulus for athletes, illustrating how a classic ERP paradigm can be made more ecologically specific while retaining the same core component logic.

The literature also includes a distinct but related set of selective-attention and orienting paradigms beyond standard oddball designs. Study [43] used exogenous and endogenous variants of the Attention Network Test (ANT), combining cue manipulations and target congruency to dissociate bottom-up and top-down orienting and analyse target-locked ERPs (e.g., P1, N1, P2, P3) across attentional modes. Study [36] used a “tunnel vision” visual discrimination task (central vs. peripheral targets) together with a pathway-weighted checkerboard pattern-reversal paradigm to bias magnocellular vs. parvocellular processing, thereby testing both spatial-attentional narrowing and low-level visual pathway sensitivity under sleep deprivation. These paradigms are methodologically important because they probe whether sleep loss affects not just generic target detection, but the balance between central/peripheral processing and stimulus-driven vs. goal-directed attentional orienting.

Another important paradigm group focused on performance monitoring and error processing, often using tasks that produce sufficient errors for robust response-locked ERP estimation. Studies [45,51,56,67,87,97] are key examples. These paradigms included stop-signal tasks (SSTs), combined flanker/Go-NoGo designs, and other speeded tasks that elicited both correct and incorrect responses, enabling analysis of ERN/Ne (error-related negativity) and Pe (error positivity), along with stimulus-locked N2/P3 measures. This family is particularly informative in sleep deprivation research because it distinguishes between two possibilities: (1) individuals become behaviourally worse but still neurally detect errors normally, or (2) sleep deprivation also blunts the brain’s internal monitoring and adaptive control systems. Studies comparing acute TSD and chronic sleep restriction (e.g., [87]) were especially valuable because they showed that “sleep loss” is not a unitary condition—different deprivation regimens can produce different error-monitoring ERP profiles.

A separate foundational family, especially in older and classic work, is the CNV warning–imperative paradigm, used in [34,62,63,92,106], and [107]. In these tasks, a warning stimulus (S1) is followed after a fixed interval by an imperative stimulus (S2), and participants prepare and execute a speeded response. The long S1–S2 interval elicits the contingent negative variation (CNV), a slow potential associated with expectancy, preparatory attention, motivation, and motor readiness. These paradigms differ from standard stimulus-locked oddball or Go/NoGo tasks because they index a prolonged preparatory state rather than a brief evoked response. Some studies quantified CNV using integrated amplitude or terminal-CNV measures across the interval, and several combined CNV with P300 or auditory evoked analyses in the same participants. This makes CNV paradigms especially useful for testing whether sleep deprivation disrupts anticipatory preparation and tonic readiness, as opposed to only post-stimulus evaluation.

The corpus also includes a substantial set of auditory sensory and evoked-response paradigms that probe lower-level or more physiological processing under sleep loss. These encompass simple auditory reaction paradigms and long-latency auditory evoked responses [94,99,107,109], often using highly repeatable tones or clicks and minimal cognitive demands. For example, [94] examined auditory ERPs in relation to sleep inertia and controlled awakenings from stage 2 sleep, focusing on the vertex N1–P2 complex rather than task-evoked cognitive control. Study [99] combined cortical auditory ERP components (including MMN/P300) with brainstem auditory evoked potentials (BAEPs), extending the sleep deprivation question across multiple levels of the auditory system. Study [109] employed repeated click-evoked long-latency responses prior to MSLT nap opportunities to compare physiological sleepiness in sleep deprivation and narcolepsy. These paradigms are important because they show that ERP sleep deprivation research is not limited to high-level cognition; it also investigates changes in basic neural responsiveness, arousal, and pre-attentive discrimination.

Several studies further employed specialised paradigms for targeting distraction, novelty, and pre-attentive processing, including mismatch and novelty designs. Study [59] used an auditory–auditory distraction paradigm in which participants performed a duration discrimination task while ignoring rare, irrelevant pitch deviants, and analysed difference waves to isolate MMN (pre-attentive deviance detection) and P3a (involuntary attention capture). This is methodologically robust because it separates automatic sensory change detection from subsequent orienting processes. Study [58] similarly implemented a novelty oddball to dissociate target P3 from novelty-related frontal positivity. Together, these paradigms clarify whether sleep deprivation primarily dampens controlled target processing, alters distractibility, or changes pre-attentive deviance sensitivity.

The reviewed studies also extend ERP methodologies into domains of emotion, social cognition, and affect regulation. Study [95] used an emotion regulation paradigm with negative and neutral IAPS images followed by instructions to maintain, distract, reappraise, or suppress emotional responses; the critical ERP measure was the late positive potential (LPP) after instruction onset, indexing the neural implementation of regulation. Study [101] used an empathy-for-pain paradigm (painful vs. non-painful scenes), analysing components such as N2, N340, and LPP and complementing them with time-frequency (theta) analysis. Studies [72,82,83] also fall partly in this affective branch by using emotional conflict, facial emotion categorisation, or affective clinical ERP frameworks. These paradigms are important because they show that sleep deprivation effects extend beyond neutral cognition into emotional salience processing, empathy, and mood-related mechanisms, which is especially relevant for psychiatric and social functioning.

Another distinct branch involves pain-evoked and sensory-affective adaptation paradigms. Study [104] used laser-evoked potentials (LEPs) with repeated noxious stimulation blocks and attention manipulations (focus, neutral, distraction) to assess not only ERP component amplitudes (e.g., temporal N1 and vertex N2/P2) but also habituation dynamics across repeated stimuli. This is conceptually different from standard one-shot ERP paradigms because the dependent variable includes change over repeated responses (habituation slope), making it well suited for testing how sleep deprivation alters sensory-affective adaptation and pain modulation.

The corpus also includes semantic learning and memory-related ERP paradigms, particularly [111], which used a paired-associate word-learning and recognition design to elicit the N400 as a marker of semantic association strength. By comparing old intact, old rearranged, and new word pairs, the paradigm dissociated genuine associative learning from repetition priming. This extends ERP sleep deprivation research beyond attention and executive function into overnight memory consolidation and semantic integration, showing that the same component-based ERP logic can be applied to sleep-dependent learning outcomes.

Several studies also adopted applied or ecologically oriented paradigms, broadening the field beyond classical laboratory tasks. Examples include hazard perception in novice drivers ([77]), driver- and military-relevant attention paradigms [43,50], and athlete-focused cognitive paradigms [56,108,115]. These tasks are important because they embed ERP measures in contexts closer to real-world safety, performance, or training demands. Although they often preserve standard ERP elements (e.g., target detection, conflict, P3), the ecological framing changes the interpretive emphasis from abstract cognitive processes to applied fatigue vulnerability and operational functioning.

Across all paradigm families, there were several shared technical and analytical features. Most studies used standard ERP pipelines: stimulus-locked (and in some cases response-locked) epochs, short pre-stimulus baselines (commonly −200 to 0 ms), EOG monitoring with ocular correction (regression or ICA), artifact rejection thresholds, and averaging over correct trials or condition-specific trial types. Electrode montages ranged from sparse midline setups to high-density arrays (64/128/256 channels), and component scoring relied on predefined time windows and electrodes informed by prior literature. Some studies used peak amplitude/latency (especially for sharper auditory or early sensory peaks), whereas others preferred mean amplitude for broader components such as P3, LPP, N2pc, or slow waves, which can be more robust under noisy, drowsy conditions. A recurring strength was the use of within-subject repeated-measures designs (rested, deprivation, recovery; sometimes multiple deprivation durations), which improved sensitivity to state-dependent ERP changes by reducing inter-individual variability. At the same time, some studies used between-group designs to reduce retest or practice confounds, especially in larger or randomised protocols.

Finally, a methodological trend in the more recent literature is the expansion of the ERP paradigm from component averaging to hybrid ERP + oscillatory/connectivity analyses, while keeping the behavioural task unchanged. Examples include time-frequency analyses in task switching and empathy paradigms [93,101], ERP–delta associations in Stroop-like competition contexts ([108]), and source-level or network connectivity analyses layered onto 2-back paradigms [103,115]. This trend is important because it preserves the stage-specific strengths of ERP paradigms while adding information about distributed neural coordination and compensatory recruitment under sleep loss.

### 3.3. ERPs Results

**Figure 3:**
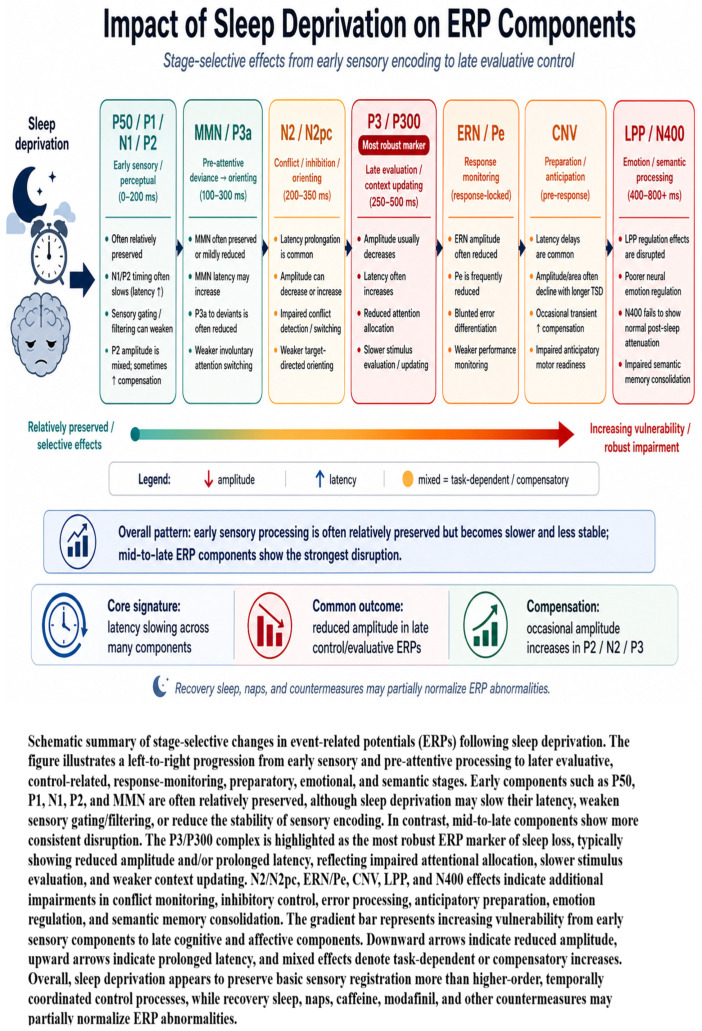
The most important findings regarding the effect of sleep deprivation on event-related potentials.

Figure 3 presents the most important findings regarding the effect of sleep deprivation on event-related potentials.

#### 3.3.1. Global Pattern: Late-Stage Vulnerability, Temporal Slowing, and Selective Compensation

The broadest cross-study finding is that total sleep deprivation (TSD) more reliably affects later-stage evaluative and control-related ERPs than early sensory registration [34,35,36,37,38,39,40,41,42,43,44,45,46,47,48,49,50,51,52,53,54,55,56,57,58,59,60,61,62,63,66,69,74,78,79,85,88,89,91,93,96,99,100,103]. P300-family components were repeatedly reduced in amplitude and/or delayed in latency across auditory oddball, Go/NoGo, stop-signal, vigilance, N-back, task-switching, and novelty paradigms, consistent with impaired attentional resource allocation, slower stimulus evaluation, and weaker updating of task-relevant representations [34,37,39,40,41,42,44,45,46,47,48,49,52,53,55,56,57,58,61,62,63,66,69,74,78,79,85,88,89,91,93,96,99,100,103,105,112,113]. A recurrent pattern was that latency effects were especially prominent in simpler oddball and repeated-testing paradigms [34,37,39,41,42,61,62,63,66,74,78,79,91,99], whereas amplitude reductions were often more robust in tasks emphasising executive control, sustained attention, or working memory [44,45,46,47,48,49,52,53,55,56,57,64,72,75,84,86,88,89,90,93,103,115]. At the same time, the literature also includes amplitude increases (e.g., P2, N2, or P3 in some tasks), typically interpreted as compensatory effort or reallocation when top-down systems are strained [39,46,52,62,96,100,109,113].

This stage-selective account is supported by multiple dissociations: early sensory/perceptual markers may remain stable while later P3 declines [39,40,41,43,47,93,98]; pre-attentive deviance detection (MMN) may be preserved while involuntary attention switching (P3a) weakens [59,99]; and early motor-preparation stages may degrade while later execution-related activity remains intact [102]. The evidence therefore points to a dynamic pattern of weakening, slowing, and compensation, not a single uniform direction of change [34,35,36,37,38,39,40,41,42,43,44,45,46,47,48,49,50,51,52,53,54,55,56,57,58,59,60,61,62,63,64,65,66,67,68,69,70,71,72,73,74,75,76,77,78,79,80,81,82,83,84,85,86,87,88,89,90,91,92,93,94,95,96,97,98,99,100,101,102,103,104,105,106,107,108,109,110,111,112,113,114,115].

#### 3.3.2. P3/P300 Complex Across Paradigms: The Most Robust ERP Marker of Sleep Loss

The P3/P300 complex was the most consistent and reproducible ERP correlate of sleep deprivation across the corpus [34,35,36,37,38,39,40,41,42,43,44,45,46,47,48,49,50,51,52,53,54,55,56,57,58,59,60,61,62,63,66,69,74,78,79,85,88,89,91,93,96,99,100,103,105,112,113]. Across auditory oddball, visual target detection, vigilance/PVT-like tasks, inhibitory-control tasks, novelty paradigms, and working-memory paradigms, sleep deprivation commonly produced reduced P3 amplitude and often prolonged P3 latency, indicating reduced attentional allocation and slower cognitive evaluation [34,37,39,40,41,42,44,45,46,47,48,49,52,53,55,56,57,58,61,62,63,66,69,74,78,79,85,88,89,90,91,93,96,99,100,103,105,112,113].

In auditory oddball paradigms, the clearest pattern was P300 latency prolongation [34,37,39,41,42,61,62,63,66,78,79,91,99]. Several studies reported substantial increases in P300 latency after acute TSD (e.g., 24 h, 36–38 h, or ~24–32 h awake), often with relatively preserved early components and variable amplitude effects [34,41,42,63]. Some studies showed selective P300 latency prolongation with little or no amplitude change [34,41,42,63,99], indicating slowed later stimulus evaluation without generalised waveform collapse; others reported both latency increases and amplitude reductions, often alongside behavioural attention decline [37,39,61,66,78,79,91]. Atypical patterns were also reported, including increased P300 amplitude despite delayed latency [62] and delayed post-recovery amplitude deterioration without a clear latency effect [38]. Portable rapid-testing paradigms (e.g., NeuroCatch) likewise detected sleep-loss effects, in one case more clearly in P300 amplitude than latency [40].

In visual vigilance and target-detection paradigms, P3 reductions and delays were common and often linked to lapses, response slowing, and variability [68,69,74,88,89,90]. Several studies further showed time-on-task and circadian modulation, with stronger P3 deterioration as fatigue accumulated within a session or during circadian vulnerability windows [69,91]. Inter-individual difference studies also found that sleep-loss-vulnerable individuals exhibited greater P3 disruption, whereas more resilient participants showed relative preservation [88,89].

In inhibitory-control and conflict paradigms, P3 effects were highly reliable but context-dependent in interpretation. Reduced NoGo-P3 or stop-P3 amplitude generally reflected impaired inhibitory evaluation and reduced control-resource allocation [35,45,52,53,55,56,64,72,75,84,86,112,113], while delayed P3 latencies indicated slower inhibitory stimulus evaluation in some but not all paradigms [35,52,53,112,113]. In some stop-signal work, P3 amplitude decreased robustly without a corresponding latency delay, suggesting weakened inhibitory engagement rather than pure temporal slowing [45]. Conversely, some paradigms reported P3 amplitude increases under TSD, interpreted as compensatory recruitment despite worsening behaviour [96,100,113].

In novelty and distraction paradigms, both target P3 and novelty-related P3 were affected [58,59]. TSD reduced and delayed novelty-related frontal P3 (novel P3/P3a-like activity), sometimes with posterior shifts or later parietal positivity increases, suggesting frontal novelty-processing impairment with compensatory posterior processing [58,59]. In auditory distraction tasks, reduced P3a amplitude to task-irrelevant deviants indicated weaker involuntary attention switching under sleep loss, often with preserved MMN [59]. One-night recovery sleep could substantially normalise novelty and target P3 in some cases [58].

#### 3.3.3. Early Sensory and Perceptual Components (P1/N1/P2): Often Preserved, but Task-Dependent Vulnerabilities Are Common

Early sensory components are often described as relatively sparse; however, the broader literature shows a mixed picture [36,39,40,41,43,47,50,63,65,68,74,80,81,85,90,93,98,99,107]. Across auditory and visual paradigms, P1/N1/P2 effects were generally smaller and less consistent than P3 effects, but they were not uniformly absent [65,68,80,85,90]. Sleep deprivation could weaken early encoding, slow early processing, or alter early attentional filtering, particularly under demanding conditions, prolonged wakefulness, or repeated testing [52,63,68,74,80,81,90,107].

In several auditory oddball and rapid auditory ERP paradigms, N100 remained stable while P300 changed markedly [39,40,41], supporting relative preservation of basic auditory registration. However, some studies found N1 latency prolongation [63,99,107], and severe deprivation could produce both delayed early auditory peaks and reduced N1/P2 amplitudes [107]. Some auditory work extended this slowing to brainstem levels (BAEP Wave I/V latency increases), indicating sleep-loss-related slowing beyond cortical processing [99].

In visual attention paradigms, early component findings were likewise mixed. Some selective-attention studies found preserved P1 and occipito-temporal N1 effects after TSD, suggesting intact early sensory gating and stimulus-driven selection [43], but still identified selective deficits in parietal N1 during endogenous orienting [43]. Visual vigilance/target detection studies often reported early attenuation with worsening performance, especially with prolonged wakefulness or repeated testing [68,74,90].

The P2/P200 component was especially heterogeneous and often informative [34,39,43,46,52,63,98,103,112]. Several studies reported increased P2 amplitude under TSD (including auditory, Go/NoGo, and working-memory contexts), interpreted as compensatory early attentional/perceptual enhancement, difficulty disengaging attention, or affective/arousal strain [39,46,52,112]. Other studies found no P2 amplitude change in simple auditory oddball paradigms [34,63], and some reported P2 reductions in working-memory or deprivation-comparison designs [103]. P2 latency was often prolonged [46,52,93,112], but some paradigms reported shortened P2 latency under TSD with incomplete normalisation after recovery [98], indicating dissociable amplitude and latency dynamics.

Sleep deprivation also affected sensory gating and attentional filtering in auditory selective-attention paradigms, where differentiation between attended and unattended stimuli was reduced, consistent with impaired top-down modulation of early perceptual stages [80,81]. Overall, early ERP effects were less reproducible than later P3 effects, but they clearly indicate that sleep loss can extend into sensory/perceptual processing depending on task structure, modality, and sleep pressure severity [65,68,80,85,90].

#### 3.3.4. MMN and Pre-Attentive Auditory Deviance Processing

A smaller yet notable set of studies examined mismatch negativity (MMN) and related deviance responses [58,59,78,85,99]. The MMN literature suggests that sleep deprivation can affect pre-attentive auditory change detection, but findings vary by paradigm [78,85,99].

Some studies reported reduced MMN amplitude or altered deviance-related negativity during prolonged wakefulness, indicating impaired automatic prediction error processing [78,85]. Others found preserved MMN amplitude but prolonged MMN latency, consistent with slowed but not abolished pre-attentive discrimination [99]. A particularly informative dissociation emerged in distraction paradigms: MMN remained unchanged while P3a to task-irrelevant deviants was reduced, indicating preserved deviance detection but weaker involuntary attentional switching [59]. This pattern fits the broader stage-selective model in which pre-attentive detection may be relatively preserved while later orienting/capture processes degrade [59,99].

#### 3.3.5. N2 and Related Control-Oriented Negativities: Conflict Detection, Inhibition, and Switching Are Reliably Disrupted

The N2 family (including frontocentral Go/NoGo N2, conflict N2, novelty-related N2, and working memory N2/N200) was a recurrent marker of sleep loss effects on executive control and conflict monitoring [39,44,45,46,47,48,49,52,53,57,64,72,73,75,82,84,86,93,96,100,103,112,113]. Across Go/NoGo, stop-signal, conflict, emotional conflict, task-switching, and N-back paradigms, studies frequently reported N2 attenuation, latency prolongation, or both [39,44,46,48,52,53,57,64,72,75,84,86,93,112,113]. These changes are generally interpreted as impaired conflict detection, weakened inhibitory engagement, and/or slower recruitment of control-related neural systems [44,52,53,64,72,75,84,86,93].

In Go/NoGo and stop-signal paradigms, N2 findings were less uniform than P3 findings but remained informative. Some studies reported smaller NoGo-N2 amplitudes under TSD, consistent with weaker inhibitory/conflict processing [52,64,86]; others found stable N2 amplitude but prolonged N2 latency, suggesting preserved monitoring magnitude but slowed processing [53,112,113]. In some stop-signal work, stop-N200 showed no significant sleep effect even when stop-P3 was strongly reduced [45]. Group-specific effects also emerged: trained athletes sometimes showed preserved N2 under TSD while controls showed reduced N2 negativity [56].

In task-switching and cognitive flexibility paradigms, N2 effects were pronounced. TSD increased N2 latency and altered N2 amplitude selectively during switch trials, indicating impaired conflict monitoring and executive control under switching demands [93]. These switch-specific N2 effects often co-occurred with later P3 alterations, supporting a staged deficit account in flexible control tasks [93].

In working memory paradigms, N2 latency prolongation was common, whereas amplitude changes were more variable [44,46,48,49,57,100,103,105]. Some studies reported increased N2 negativity under TSD (interpreted as compensatory conflict monitoring), especially in difficult or incongruent conditions [100,103,105]; others found no significant N2 amplitude effects despite latency slowing [48,49,57]. Longer deprivation (e.g., 30 h vs. 24 h) could shift the pattern toward stronger impairment, with increased N2 negativity coexisting with larger P3 reduction and P3 latency prolongation [103].

In conflict and emotional conflict paradigms, N2 alterations indicated impaired detection/resolution of competing information, including emotionally salient conflict [72,73,82]. Some studies further showed that incentives modulated N2 and later ERP effects, suggesting that motivational context can partly reshape sleep-loss-related control deficits [73].

A few specialised visual attention studies also implicated related negativities: for example, the N2pc (lateralised target-orienting index) became less negative after TSD and correlated with accuracy decline, indicating weaker target-directed orienting [96]. This extends sleep loss effects beyond generic frontocentral N2 to selective-attention-orienting mechanisms [96].

#### 3.3.6. Inhibitory Control and Executive Control: Dissociation of Early and Late Control Stages

Go/NoGo and stop-signal studies collectively showed that sleep deprivation impairs inhibitory control, but with a meaningful dissociation between early monitoring/conflict stages (N2-related) and later inhibitory evaluation stages (P3-related) [35,45,52,53,55,56,64,72,75,84,86,112,113]. The most robust effect across these paradigms was reduced NoGo-/stop-P3 amplitude, often with P3 latency prolongation, indicating weakened late-stage inhibitory processing and reduced control-resource allocation [35,45,52,53,55,56,64,86,112,113].

At the same time, N2 in inhibitory paradigms was more heterogeneous: it could be reduced [52,56,64,86], delayed [53,112,113], preserved [45], or partially restored by countermeasures (e.g., caffeine improving some NoGo-N2 indices after TSD) [112]. This variability supports the view that N2 does not simply collapse under sleep deprivation; rather, early control monitoring may be preserved in magnitude but slowed, selectively reduced, or modulated by participant profile and intervention [45,53,56,112]. The executive control literature therefore reinforces a central synthesis: sleep deprivation reliably degrades late inhibitory evaluation (P3), while earlier conflict-monitoring processes (N2) show more context-dependent vulnerability [35,45,52,53,56,64,72,75,84,86,112,113].

#### 3.3.7. Error Monitoring and Performance Monitoring (ERN/Ne, Pe): Weakened Frontal Monitoring and Reduced Error Differentiation

Response-locked ERP studies provided strong evidence that sleep deprivation impairs performance monitoring, especially frontal error-detection systems indexed by ERN/Ne and, in many cases, later Pe [45,51,56,67,87,97].

Across Flanker, Go/NoGo, and conflict tasks, sleep deprivation often led to reduced ERN amplitude, indicating weaker early error-detection signalling [51,56,67,87,97]. In some studies, ERN latency remained unchanged, suggesting preserved timing but reduced neural magnitude of error detection [51]. Sleep deprivation also altered monitoring dynamics over time: rested controls showed habituation/adaptation of ERN across accumulating errors, whereas sleep-deprived participants often did not [51].

Pe findings were more variable but generally pointed to impaired later evaluative/error-awareness processing [45,51,67,87]. Some studies reported reduced Pe amplitude under TSD with preserved Pe latency [45], while others found more selective or performance-dependent Pe effects (e.g., larger Pe in poor-performing sleep-deprived participants), interpreted as perseverative or affectively loaded error processing [51].

A particularly strong result came from conflict/action monitoring work showing that sleep deprivation not only reduced error-related negativity but also blunted the normal graded differentiation between full errors, partial errors, and correct responses, especially in incongruent trials [97]. This indicates a loss of neural sensitivity to response correctness and error severity, not merely noisier behaviour [97]. Participant characteristics also mattered: trained athletes sometimes showed relative preservation of ERN under TSD compared with controls [56].

#### 3.3.8. Working Memory ERPs: Staged Disruption of the P2–N2–P3 Complex

Working memory studies consistently implicated the P2–N2–P3 complex, but the pattern supports a staged model rather than a single global deficit [44,46,47,48,49,57,98,100,103,105,115]. The most reliable result was P3 amplitude reduction (especially at frontal/central sites), indicating weakened top-down resource allocation during updating and decision processes [44,46,48,57,103,105,115]. Some studies also found P3 latency prolongation, but latency effects in working memory were less consistent than amplitude reductions [44,46,48,49,57,103,105]. Therefore, in demanding updating contexts, P3 amplitude is often the more stable marker of dysfunction [44,46,47,48,49,57].

Several studies demonstrated topographic and compensatory complexity. In one visual working memory study, TSD reduced frontal/central P3 but increased parietal P3, interpreted as compensatory parietal recruitment in response to weakened frontal executive systems [47]. Another study found loss of normal right hemisphere P3 dominance in spatial working memory after TSD [48]. A multimodal study further linked reduced frontal P3 amplitude during a spatial 2-back task to decreased beta-band connectivity (especially frontal–occipital), alongside smaller compensatory connectivity increases [115].

Earlier components in working memory showed mixed but interpretable changes. P2 often increased and/or slowed under TSD in some paradigms [44,46,98], but was unchanged or reduced in others [47,103]. N2 latency was frequently prolonged [44,46,48,57], whereas amplitude could increase (compensation), decrease, or remain stable depending on task load and design [46,48,49,57,100,103,105]. A particularly informative set of studies comparing 24 h vs. 30 h TSD suggested that with increasing sleep pressure, early compensatory changes (e.g., more negative N2) may coexist with stronger later-stage failure (larger P3 reduction and longer P3 latency), indicating that compensation has limits [103].

Pharmacological manipulations also clarified stage specificity. In modafinil/caffeine/placebo crossover work, modafinil better preserved P3 amplitude and shortened N2/P3 latencies relative to placebo (and often caffeine), suggesting stronger buffering of later updating/allocation processes [105]. Caffeine showed clearer effects on earlier components (P2/N2) than on P3 in some inhibitory paradigms [112].

#### 3.3.9. Selective Attention, Orienting, Visual Search, and Sensory–Motor Preparation

Studies of selective attention and visual processing broadly support the pattern of relative preservation of early sensory components alongside degradation of later evaluative stages, while also revealing important exceptions linked to top-down orienting and pathway-specific effects [36,43,50,68,74,90,93,96,102].

In selective-attention paradigms (including endogenous/exogenous orienting tasks), early P1 and occipito-temporal N1 effects were sometimes preserved after TSD [43], indicating intact basic sensory gating and stimulus-driven selection. However, sleep deprivation could selectively reduce parietal N1 cueing effects in endogenous attention [43], indicating impaired top-down spatial orienting. Mid/late effects in the same paradigms often included increased P2 and reduced P3 [43].

Visual search paradigms offered a compelling fractionation. In one 36 h TSD visual search study, N2pc (target orienting) became less negative and correlated with accuracy decline, indicating weakened selective orienting [96]. At the same time, P3 amplitude increased, interpreted as compensatory recruitment of later context-updating/control processes [96]. This dissociation (weaker early orienting but stronger later P3) is a recurrent compensatory motif [96].

Motor preparation measures further refined this stage-specific picture. In a stimulus–response compatibility task, the earlier sensory-integration/motor-preparation stage indexed by s-LRP lost its normal compatibility differentiation after TSD, whereas the later execution-related r-LRP remained largely unaffected in amplitude and latency [102]. Thus, TSD impaired the stage where sensory evidence is translated into motor preparation while sparing later response execution-related activation [102].

Visual pathway-weighted tasks also revealed selective early vulnerability: while magnocellular-related early ERPs were relatively stable, parvocellular P100 latency could be prolonged under TSD [36], indicating specific slowing in detailed/sustained visual processing. This qualifies the common “early processing spared” claim and suggests pathway-dependent vulnerability [36].

#### 3.3.10. Auditory Processing, AEPs, and Sleep Inertia-Related ERP Changes

Auditory ERP/AEP studies showed one of the most consistent latency findings in the literature: sleep deprivation often produces auditory processing slowing, reflected in latency increases across cortical and, in some cases, brainstem auditory responses [34,39,40,41,42,61,62,63,78,79,91,99,107].

Acute TSD frequently prolonged auditory P300 latency [34,37,39,41,42,61,62,63,66,78,79,91,99], and in some studies also prolonged MMN latency [99], even when amplitudes remained relatively stable [34,41,42,63,99]. More severe deprivation could additionally delay early cortical AEP peaks (P1/N1) and reduce N1/P2 amplitudes [107]. Some studies extended this slowing to the BAEP, with prolonged Wave I and/or Wave V latencies, indicating effects through early auditory pathways [99].

Auditory amplitude patterns were not unitary. In some sleepiness-focused work, long-latency click-evoked amplitudes (e.g., N1–P2, P2–N2) increased after deprivation in healthy controls, whereas clinical/pathological sleepiness groups showed more complex state-dependent patterns [109]. These findings suggest that “sleepiness-related” auditory ERP changes depend strongly on physiological context (acute TSD vs. pathological sleepiness) and on whether latency or amplitude is measured [99,107,109].

A related line of work on sleep inertia (post-awakening hypoarousal) showed reduced N1–P2 amplitude immediately after awakening relative to pre-sleep wakefulness, with stronger/persisting attenuation after recovery sleep with SWS rebound and altered scalp topography [94]. Here, prior sleep architecture modulated post-awakening cortical responsiveness, and N1 latency was prolonged after nocturnal awakenings while P2 latency was relatively stable [94]. These findings complement TSD results by showing that awakening state and prior sleep composition also shape auditory ERP responsiveness [94].

#### 3.3.11. CNV and Preparatory Processing: Robust Vulnerability of Anticipatory Brain States

CNV studies provided strong evidence that sleep deprivation impairs not only stimulus evaluation but also anticipatory preparation and motor readiness [34,62,63,92,107]. Across CNV paradigms, TSD altered CNV timing and/or amplitude, often alongside reaction-time slowing, indicating degraded preparatory cortical activation [34,62,63,92,107].

Some studies reported CNV latency delays (e.g., delayed early CNV marker M1 or later marker M2) with slowed RT but limited amplitude change, suggesting slowed preparatory processing [34,63]. Others found broader effects, including prolonged CNV latencies plus increased CNV amplitudes, interpreted as compensatory effort or altered preparatory activation under strain [62].

A second line of CNV research under longer wakefulness (36–48 h) showed a more dramatic pattern of CNV amplitude/area reduction [92,107]. CNV area or maximum amplitude declined sharply over TSD, often with pronounced drops during the circadian low, increased inter-individual variability, and in some cases polarity reversals toward CPV-like forms [92]. These studies also reported delayed and reduced auditory warning-tone responses and slowed RT [107], indicating broad weakening of the preparatory response system [92,107].

#### 3.3.12. Novelty Processing, Involuntary Attention Capture, and Distraction

Specialised novelty and distraction paradigms showed that sleep deprivation does not simply increase distractibility; rather, it can weaken involuntary attention capture while preserving some earlier deviance processing [58,59].

In auditory distraction tasks, both total and partial sleep deprivation reduced P3a amplitude to task-irrelevant deviants while MMN remained unchanged, indicating preserved pre-attentive deviance detection but reduced reflexive orienting [59]. Behaviourally, this often corresponded to a reduced distraction cost, especially after total deprivation [59].

Novelty oddball paradigms revealed more extensive changes: TSD reduced and delayed frontal novel P3, often with a topographic shift away from frontal dominance and increased later parietal positivity [58]. Target P3 was also reduced and delayed [58]. This pattern suggests degraded novelty and target evaluation with possible posterior compensatory processing [58]. After one night of recovery sleep, novelty and target P3 measures could largely normalise [58].

#### 3.3.13. Emotion, Motivation, Empathy, Pain, and Semantic Processing: Late Positive and Domain-Specific ERP Alterations

ERP studies of affective and motivational processing showed that sleep deprivation alters neural responses beyond classic attention/executive domains [72,73,82,83,95,101,104,111].

In emotion regulation paradigms, a key result was loss of normal modulation of the LPP. In rested participants, distraction and reappraisal reduced LPP amplitude relative to passive maintenance of emotion, reflecting successful regulation. In sleep-deprived participants, these strategies failed to reliably reduce the LPP, indicating impaired implementation of neural emotion regulation [95]. Suppression did not reduce the LPP in either group in that study [95].

In emotional conflict and affective-control tasks, sleep deprivation altered late positive activity (P3/LPP-like responses) and N2-related conflict signals, consistent with less efficient processing of emotionally incongruent information [72,82]. Incentives and motivational context could reshape ERP effects, suggesting that reward significance can partially buffer or modify control-related deficits under sleep loss [73].

In empathy-for-pain paradigms, TSD reduced early-to-mid affective resonance markers (e.g., N2, N340), particularly for painful stimuli, while later LPP was relatively preserved [101]. Time–frequency analyses further showed reduced theta power (200–500 ms), and in the sleep-deprived state this reduction correlated with pain/unpleasantness ratings [101].

In pain-evoked laser ERP studies, sleep deprivation produced a notable dissociation: subjective pain and unpleasantness increased, yet vertex P2 amplitude was reduced [104]. The mechanistic explanation was enhanced P2 habituation after TSD rather than globally reduced nociceptive processing; distraction abolished this TSD-related habituation effect [104]. N1 and N2 habituation did not show comparable sleep-condition effects [104].

In semantic memory consolidation, overnight wakefulness prevented the normal post-sleep attenuation of N400 to previously learned semantic pairs, indicating impaired sleep-dependent consolidation of specific associations [111]. Thus, ERP effects of sleep deprivation extend to offline memory consolidation processes, not just online performance [111].

#### 3.3.14. Topography, Lateralisation, and Source/Network-Level Findings

Beyond amplitude and latency shifts, several studies reported topographic and lateralisation changes, indicating that sleep deprivation alters the spatial organisation of neural processing [36,43,47,48,58,85,103,115].

Topographic redistribution was seen in multiple paradigms. Sleep deprivation reduced frontal novelty P3 and shifted processing toward posterior positivity in novelty tasks [58], and in working memory it sometimes reduced frontal/central P3 while increasing parietal P3, consistent with compensatory posterior recruitment [47]. In spatial working memory, TSD weakened or abolished normal right hemisphere dominance of P3 amplitude [48].

Regional specificity also appeared in component vulnerability: parietal N1 deficits emerged selectively in endogenous attention [43]; parvocellular P100 latency was selectively prolonged while magnocellular-related responses were preserved [36]; and frontal/central sites often showed stronger N2/P3 degradation in executive tasks [46,48,57,93,115]. These findings argue against a uniform dampening account and instead support selective reshaping of cortical processing priorities under fatigue [36,43,47,48,58].

A smaller subset of studies used source-level or connectivity analyses, which generally implicated distributed frontocentral and parietal attention/control systems [85,103,108,115]. Source/effective connectivity findings suggested a combination of compensation and failure: some frontal pathways or regional activity increased under TSD, while key control network connections (e.g., insula–ACC pathways; frontal–occipital beta connectivity) weakened [103,115]. In one study, Stroop incongruent P3 mediated the relation between TSD-induced Stroop RT change and later performance, with delta-band activity in the P3 window differentiating groups [108]. These multimodal findings strengthen the interpretation that ERP changes map onto behaviourally meaningful network-level reorganisation [103,108,115].

#### 3.3.15. Latency Effects as a General Signature of Slowed Neural Processing

Across many paradigms, sleep deprivation produced latency prolongation in key ERP components, especially P2, N2, and P3, and in some auditory and preparatory responses [34,37,39,41,42,44,46,48,52,53,57,61,62,63,66,69,74,78,79,91,93,99,103,105,107,112,113]. These latency shifts support the interpretation that sleep-deprived individuals require more time for stimulus evaluation, conflict detection, response selection, or preparatory processing [34,44,46,52,53,63,93,99,107].

Latency effects were particularly clear in repeated-testing and prolonged-wakefulness designs, where progressive slowing often paralleled behavioural deterioration and circadian vulnerability [69,74,91,92]. In some paradigms, latency effects were more stable than amplitude effects [34,41,42,63,99], potentially making them less sensitive to inter-individual differences in absolute EEG amplitude or electrode montage. However, latency measures were not uniformly reported across studies [64,65,66,67,68,69,70,71,72,73,74,75,76,77,78,79,80,81,82,83,84,85,86,87,88,89,90,91], which limits direct comparison. Still, the cumulative evidence strongly supports temporal slowing of neural processing as a core electrophysiological consequence of sleep deprivation [34,35,36,37,38,39,40,41,42,43,44,45,46,47,48,49,50,51,52,53,54,55,56,57,58,59,60,61,62,63,64,65,66,67,68,69,70,71,72,73,74,75,76,77,78,79,80,81,82,83,84,85,86,87,88,89,90,91,92,93,94,95,96,97,98,99,100,101,102,103,104,105,106,107,108,109,110,111,112,113,114,115].

#### 3.3.16. Recovery Sleep, Naps, Pharmacological/Behavioural Countermeasures, and Reversibility

ERP countermeasure and recovery studies indicate that sleep-deprivation-related electrophysiological impairment is often partially reversible, but recovery is component- and time-dependent [34,35,38,44,53,57,58,62,63,98,105,112,113].

Short naps produced measurable benefits in several studies. A 30 min nighttime nap during TSD reduced P300 latency, improved CNV latency, and improved RT [34]. A 2 h nap improved inhibitory ERP and behavioural performance over subsequent hours, although zaleplon introduced a short-lived post-awakening cost (e.g., temporarily longer NoGo-P3 latency) before later benefits [35]. In another study, a 1 h nap after TSD shortened P3 latency and increased N2 amplitude even without clear immediate behavioural recovery, illustrating ERP sensitivity to early restoration [113].

By contrast, short recovery periods were not always sufficient. A 110 min recovery interval after ~24 h awake did not produce reliable immediate P300 restoration in one study, and continued wakefulness afterward was associated with later P300 amplitude decline [38].

One full night of recovery sleep often improved ERP amplitudes and latencies (e.g., working memory N2/P3 and novelty P3 measures), but normalisation was sometimes incomplete after severe TSD [44,53,57,58,98]. Some studies reported partial recovery with persistent residual latency abnormalities [53], whereas others found near-complete normalisation of novelty and target P3 after one night [58]. In object working memory, P2 amplitude normalised after 8 h recovery sleep while P2 latency remained shortened, demonstrating distinct recovery time courses for amplitude vs. latency [98].

Pharmacological and behavioural countermeasures also modulated ERP effects. Modafinil generally reduced or prevented TSD-related latency slowing (P300, CNV, N2/P3) and better preserved P3 amplitude than caffeine in some paradigms [63,105]. Caffeine often had clearer effects on earlier components (P2/N2) than on later P3 [112]. Zaleplon showed a biphasic inhibitory ERP profile [35], and meditation training reduced susceptibility of P300/CNV measures to subsequent TSD in one study, although the study’s unusual amplitude patterns warrant caution [62].

#### 3.3.17. ERP–Behaviour Relationships, Individual Differences, and Translational Relevance

Across paradigms, ERP changes were frequently associated with behavioural impairments, including slower RTs, increased lapses, more errors, poorer inhibition/conflict resolution, and greater response variability [34,61,69,74,88,89,90,96,99,100,108]. In vigilance and attention tasks, reduced P3 amplitude and delayed P3/N2 latencies often tracked worsening performance [69,74,88,89,90]; in visual search, reduced N2pc correlated with accuracy decline [96]; in auditory paradigms, increased P300 latency correlated with poorer auditory discrimination [99]; and in some working memory tasks, larger compensatory P3 amplitudes were associated with faster RTs or better accuracy [100].

Several studies also emphasised individual differences in vulnerability. Participants more susceptible to sleep deprivation showed larger ERP disruptions (especially P3 deterioration), whereas resilient individuals maintained more stable neural responses despite sleep loss [88,89]. This has practical implications for operational contexts such as healthcare, driving, and shift work [50,64,88,89].

Importantly, ERP–behaviour relationships were not always one-to-one. In some cases, ERP abnormalities appeared even when behavioural performance was only mildly impaired [64,110], or ERP recovery appeared before behavioural recovery (e.g., after naps) [113]. This supports a key methodological conclusion: ERPs are often more sensitive than behaviour for detecting stage-specific degradation, compensation, and early recovery under sleep loss [110,112,113].

#### 3.3.18. Risk of Bias Assessment

The ROBINS-I bias risk assessment is presented in Table 2, and the RoB-2 bias risk assessment is presented in Table 3.

#### 3.3.19. Synthetic Summary of the Evidence

Across the 82 included studies, the evidence did not support a single uniform ERP signature of sleep deprivation. Instead, the findings converged on a stage-specific pattern: sleep deprivation most consistently affected mid-to-late cognitive and control-related components, especially the P300/P3 family, N2/P2, CNV, and response-monitoring components such as ERN/Ne and Pe. These effects were most often expressed as prolonged latency, reduced amplitude, or reduced differentiation between task-relevant and task-irrelevant conditions.

The most robust synthesis finding was that sleep deprivation impairs late stimulus evaluation, attentional allocation, and context updating. This conclusion is supported by repeated reports of P300/P3 latency prolongation and/or amplitude reduction across oddball, Go/NoGo, working memory, vigilance, conflict, and decision-making paradigms. Therefore, P300/P3 appears to be the most informative ERP marker of sleep-loss-related cognitive inefficiency. However, the direction of amplitude change was not uniform in all studies. In some high-demand or compensatory contexts, P3 amplitude increased or shifted topographically, suggesting that increased amplitude should not automatically be interpreted as improved function; rather, it may reflect compensatory recruitment under increased neural effort.

Early sensory and pre-attentive components showed a different pattern. Components such as P1, N1, MMN, and P50 were often relatively preserved, indicating that sleep deprivation does not invariably abolish initial sensory registration. Nevertheless, these early processes were not completely resistant. Several studies reported latency slowing, reduced phase locking, impaired sensory gating, or weakened attentional modulation. Thus, the best-supported conclusion is not that early processing is unaffected, but that it is less consistently disrupted than later evaluative and control-related processing.

Executive control and response-monitoring findings provide a second major synthesis conclusion. Sleep deprivation commonly reduced the efficiency of inhibition, conflict monitoring, preparatory attention, and post-error evaluation, as reflected in altered N2, CNV, ERN/Ne, and Pe responses. These effects were particularly evident in tasks requiring sustained attention, working memory, Go/NoGo control, flanker interference, or adaptive performance monitoring. The pattern suggests that sleep loss weakens top-down control and the ability to maintain stable task goals over time.

A further cross-study finding was the dissociation between behavioural and electrophysiological outcomes. In several studies, ERP abnormalities were present despite modest, inconsistent, or absent behavioural impairment. This indicates that ERPs may detect covert neural inefficiency before overt performance breakdown becomes obvious. Conversely, when behavioural impairment was clear, ERP changes often clarified whether the deficit reflected slowed stimulus evaluation, reduced attentional allocation, impaired response inhibition, or weakened error monitoring.

Finally, recovery sleep, naps, caffeine, modafinil, and other countermeasures generally produced partial rather than complete normalisation. Recovery was component-specific: behavioural performance could improve while some ERP abnormalities persisted, and amplitude and latency measures did not always recover at the same rate. This supports the conclusion that sleep-loss effects are not simply reversed uniformly by short recovery opportunities but depend on the neural process indexed by each ERP component.

To make the synthesis explicit, Table 4 summarises the strength and interpretation of evidence by ERP domain rather than by individual study. This table is intended to complement Table 1, which provides study-level details.

## 4. Discussion

The aim of this review was to clarify how sleep deprivation alters event-related potentials and what these changes reveal about the temporal stages of cognitive and affective processing. Across the included studies, the effects of sleep deprivation were not uniform across ERP components, tasks, or recovery conditions. Instead, the evidence indicates a stage-specific pattern: early sensory and pre-attentive processing is often relatively preserved, although sometimes slower or less stable, whereas mid-to-late components related to attention allocation, stimulus evaluation, inhibitory control, performance monitoring, and emotional regulation are more consistently disrupted.

To make these findings easier to interpret, the Discussion is organised around the main processing stages and functional themes emerging from the reviewed literature. First, we discuss the P300/P3 family as the most consistent ERP marker of sleep loss effects. Second, we examine early sensory and pre-attentive components, which show selective rather than global vulnerability. Third, we consider mid-latency and executive control components, including N2, P2, CNV, ERN/Ne, and Pe. Fourth, we address affective, memory-related, and task-specific ERP findings. Fifth, we discuss the dissociation between behavioural performance and ERP abnormalities. Finally, we consider recovery, naps, countermeasures, methodological limitations, and implications for future research.

### 4.1. Overall Pattern of ERP Changes After Sleep Deprivation

Overall, the reviewed evidence supports the view that sleep deprivation produces a selective disruption of information processing rather than a uniform reduction in all ERP responses. The most consistent abnormalities occurred in components reflecting attentional allocation, stimulus evaluation, cognitive control, and response monitoring. By contrast, earlier sensory responses were often preserved, although some studies reported delayed latencies, reduced filtering, impaired phase locking, or weaker attentional modulation. This pattern suggests that prolonged wakefulness mainly compromises the stability, efficiency, and coordination of neural processing, especially when tasks require sustained attention, working memory, inhibition, or adaptive control.

### 4.2. P300/P3 as the Most Consistent Marker of Sleep Loss Effects

The P300/P3 family emerged as one of the most consistent electrophysiological markers of sleep deprivation effects. These findings are best interpreted as evidence of impaired late-stage stimulus evaluation, context updating, and controlled resource allocation rather than as a simple, nonspecific slowing of neural processing. Within the context-updating framework, P3 amplitude scales with task relevance and subjective probability, whereas P3 latency reflects stimulus evaluation time [30,116,117]. This distinction is important for interpreting the two main effects observed after sleep deprivation: prolonged P3 latency suggests slower stimulus evaluation and categorisation, whereas reduced P3 amplitude suggests weaker or less stable allocation of processing resources to task-relevant information [30,118].

This amplitude–latency distinction also explains why sleep deprivation can affect electrophysiological processing even when behavioural performance is only modestly impaired. Prolonged wakefulness may delay the evaluation of a stimulus without producing a proportional change in overt response time, or it may reduce P3 amplitude without completely eliminating task performance. Thus, P3 abnormalities provide a sensitive marker of sleep-loss-related cognitive disruption because they reveal changes in the timing, strength, and reliability of late evaluative processing that may not be fully captured by accuracy or reaction time measures [30,117,118].

The centroparietal P3/CPP literature further supports this interpretation. P3-like activity has been linked to evidence accumulation during decision formation, suggesting that prolonged P3 latency under sleep deprivation may reflect slower evidence accumulation or delayed threshold attainment, whereas reduced amplitude may reflect weaker, noisier, or less coordinated decision-related activity [119,120,121]. This framework also helps explain why P3 deficits are often more pronounced in tasks involving high working-memory load, conflict, inhibition, or sustained attention: these conditions require stable top-down support, and late centroparietal activity appears especially vulnerable when arousal and control resources are degraded by sleep loss [119,120,121].

The reviewed findings also indicate that sleep deprivation reduces the functional specificity of P3-family responses. Under rested conditions, P3 activity typically differentiates between targets and nontargets, Go and NoGo trials, expected and unexpected events, or task-relevant and task-irrelevant stimuli. After sleep deprivation, this differentiation is often weakened, suggesting that the sleep-deprived brain does not merely generate smaller P3 responses but becomes less able to assign distinct electrophysiological signatures to different cognitive events. This pattern is consistent with reduced context specificity and degraded network integration in the P3 time window [118,122,123].

Topographic changes in P3 activity should therefore not be interpreted as simple amplitude reductions. Source localisation and multimodal studies indicate that visual P3 activity depends on distributed frontoparietal systems [122,124]. Accordingly, posterior redistribution of P3-family activity after sleep deprivation may reflect altered network weighting or compensatory reconfiguration when frontal control contributions weaken, rather than a uniform loss of P3 generation [122,124]. Similarly, occasional increases in P3 amplitude should not be interpreted as improved processing. Under high task demand, larger P3 responses may reflect compensatory effort or inefficient over-recruitment of evaluative resources rather than enhanced cognitive function [30,117,121].

The NoGo-P3 and stop-related P3 findings extend this interpretation to inhibitory-control paradigms. Go/NoGo and stop-signal ERP studies support the view that NoGo/stop P3 reflects late control-related processing rather than a purely motor phenomenon [125,126,127]. Therefore, delayed or reduced NoGo-P3 after sleep deprivation suggests impaired recruitment of late inhibitory-control mechanisms. This interpretation is strengthened by findings of reduced Go–NoGo P3 differentiation, which indicate that sleep loss weakens the electrophysiological separation between action execution and action withholding. Such effects are difficult to explain by motor slowing alone and instead point to impaired allocation of late control resources [125,126,127].

### 4.3. Early Sensory and Pre-Attentive Processing: Relative Preservation with Selective Vulnerability

The reviewed findings suggest that early sensory and pre-attentive ERP components are generally more resistant to sleep deprivation than later evaluative and control-related responses, but they are not entirely unaffected. Early auditory and visual components such as N1, P1, MMN, and P50 often remained detectable after sleep loss, indicating that basic sensory registration is usually preserved. However, several studies also reported delayed latencies, reduced attentional modulation, weaker sensory gating, altered MMN responses, or poorer temporal consistency. Thus, the most accurate interpretation is not that early processing is fully spared, but that it shows relative preservation with selective vulnerability, especially when early sensory activity depends on attention, temporal precision, filtering, or predictive comparison.

This interpretation is consistent with the broader ERP literature showing that early components are not simple markers of sensory input strength. Visual P1 and N1 reflect both sensory encoding and rapid attention-dependent gain control. P1 has been linked more closely to early sensory facilitation at attended locations, whereas N1 has been associated with orienting toward task-relevant stimuli and the discrimination of relevant perceptual features [29,128,129]. Therefore, preserved P1 or N1 amplitudes after sleep deprivation should not be taken to mean that early processing is completely normal. Rather, basic registration may remain intact while the attentional amplification layered onto early sensory activity becomes weaker, slower, or less selective.

A similar interpretation applies to early auditory attention effects. Auditory selective-attention research shows that N1 enhancement to attended stimuli depends on task structure, stimulation rate, and endogenous selection processes, and that attended–unattended differences reflect more than a purely exogenous sensory response [130,131,132]. Accordingly, reduced attended–unattended differentiation in the N1 window after sleep deprivation is better interpreted as impaired early top-down signal enhancement than as a failure of auditory sensory registration itself. In this sense, sleep loss appears to degrade the efficiency with which attention biases early encoding, while leaving the basic capacity to generate early auditory responses relatively preserved.

Latency findings further support this selective-vulnerability account. The broader ERP literature shows that early-component latency can vary with sensory discriminability, attentional set, and temporal uncertainty even when amplitude remains relatively stable [29,133]. Therefore, findings such as preserved N1 amplitude but delayed N1, MMN, or visual P1 responses suggest that sleep deprivation may slow the temporal unfolding of early sensory–attentional operations without necessarily reducing peak response magnitude. Mechanistically, the response can still be generated, but cortical engagement becomes less temporally efficient, which may contribute to slower downstream stimulus evaluation and response preparation.

The MMN findings fit this same pattern. MMN is generally understood as an index of automatic deviance detection based on short-term regularity formation and comparison between expected and incoming auditory input [134,135,136]. The common findings of preserved MMN with reduced later P3a/P3b responses suggests that automatic deviance registration can remain relatively intact while subsequent orienting and controlled allocation of attention are more vulnerable to sleep loss. However, MMN should not be treated as uniformly preserved. Because MMN amplitude and latency depend on deviant magnitude, regularity complexity, and the fidelity of the sensory-memory trace, attenuation or latency prolongation under more demanding conditions is mechanistically plausible [134,135,136]. Thus, mixed MMN findings are better interpreted as boundary condition effects than as contradictions: automatic comparison processes are relatively resilient, but they can become slower or less precise when sensory discrimination demands, sleep pressure, or task complexity increase.

The P50 findings provide another example of selective early vulnerability. Paired-click P50 suppression is usually interpreted as an early inhibitory filtering mechanism that reduces cortical responses to redundant input. Broader sensory-gating research links this process to hippocampal, temporal, and prefrontal circuitry, with important cholinergic and inhibitory contributions [137,138,139,140]. Within this framework, preserved S1 responses with weaker S2 suppression after sleep deprivation indicate that the initial stimulus can still be encoded, but the inhibitory gate that normally filters redundant input is weakened. This suggests that sleep loss affects early filtering and sensory control more than basic sensory registration itself, potentially increasing vulnerability to distractibility and redundant sensory load [139,140].

The visual findings also support a task-dependent rather than globally suppressive effect of sleep deprivation. P1 and N1 are strongly modulated by attended features, spatial focus, perceptual demand, and stimulus ambiguity, and different task configurations can affect these components in different ways [129,130,141,142]. Therefore, the absence of a general P1/N1 collapse does not mean that early visual processing is unaffected. Instead, sleep deprivation appears to interact with specific gain-control operations, including spatial attention, feature selection, perceptual ambiguity, and emotional or threat-related salience. Reported parvocellular-weighted latency effects and emotion- or ambiguity-sensitive P1/N170 changes are consistent with this interpretation, because they reflect selective modulation of early visual pathways rather than uniform degradation of sensory processing.

Finally, amplitude increases in early components under sleep deprivation should be interpreted cautiously. In attention and sensory-gating research, larger early ERP amplitudes can reflect increased orienting, altered excitability, reduced inhibitory filtering, or changes in arousal state rather than better perceptual processing [131,133,140,141]. This is particularly relevant for auditory amplitude increases after sleep loss, which may indicate altered sensory gain or reduced filtering rather than enhanced perception. Because cholinergic tone and arousal regulation influence early sensory gain and gating, sleep deprivation may produce larger early responses in some contexts while simultaneously degrading selective filtering and later cognitive control [138,139,143].

### 4.4. P2 and N2: Intermediate Markers of Selection, Conflict, and Compensatory Control

The P2 and N2 findings are best interpreted as evidence that sleep deprivation disrupts intermediate stages of processing that link early perceptual analysis with later controlled evaluation and action regulation. These components are particularly sensitive to task architecture because they do not index single, isolated operations. Rather, they reflect temporally adjacent processes such as task-relevance evaluation, context matching, perceptual mismatch detection, conflict monitoring, and early response updating. This functional heterogeneity explains why P2 and N2 effects under sleep deprivation are less uniform than P3 effects, while still providing important evidence of impaired cognitive control.

The anterior P2, often described as frontal selection positivity or P2a, has been linked to task relevance and stimulus evaluation rather than to simple sensory or motor processing [144]. Related work in context-dependent working memory control suggests that activity in the P2 time range may reflect early prefrontal context updating, preceding later posterior P3b-related updating [145]. Within this framework, increased P2 amplitude after sleep deprivation may reflect compensatory upregulation of early task-relevance evaluation or context-setting, especially when participants attempt to maintain performance under fatigue. Conversely, reduced P2 amplitude may indicate diminished efficiency of the same early evaluative process when compensatory recruitment is insufficient. Therefore, larger P2 responses should not be interpreted automatically as improved processing; they may instead reflect increased effort, altered gain, or inefficient early recruitment of control resources [144,145,146,147,148].

The N2 findings require a similarly cautious interpretation. A major review by Folstein and Van Petten showed that “N2” is not a single component, but a family of anterior and posterior negativities whose functional meaning depends strongly on task demands [149]. In some paradigms, N2 reflects conflict monitoring or response competition; in others, it reflects perceptual mismatch, template violation, or attentional selection. This helps explain why N2 amplitude can increase, decrease, or remain stable across sleep deprivation studies. Amplitude changes may reflect different balances between compensatory conflict monitoring, reduced control efficiency, and paradigm-specific mismatch processing rather than a single uniform sleep-loss mechanism [149].

By contrast, N2 latency prolongation appears more mechanistically consistent. Go/NoGo and interference studies indicate that frontocentral N2 is strongly associated with response conflict and high-competition response contexts, rather than inhibition alone [150,151]. Related work links frontocentral N2 to anterior cingulate and medial-frontal monitoring systems involved in detecting conflict and signalling the need for control [151,152]. Therefore, delayed N2 after sleep deprivation suggests slower recruitment of medial-frontal control mechanisms, even when the amplitude of the monitoring response is relatively preserved. In this sense, latency slowing may be a more reliable indicator of sleep-loss-related impairment than amplitude direction.

The inhibitory control literature further supports separating N2 from later P3-family effects. Early Go/NoGo work associated larger or delayed NoGo-N2 responses with inhibitory demands and false-alarm tendencies, but also showed that modality and scalp distribution strongly influence the observed pattern [153]. Later studies clarified that N2 most likely reflects an earlier non-motor control stage, such as conflict monitoring or recognition of the need for control, whereas P3 is more closely tied to later evaluative and response-related processing [127,154]. This distinction is useful for interpreting sleep deprivation findings in which N2 increases while P3 decreases: the sleep-deprived brain may still detect conflict or even recruit compensatory early monitoring but fail to translate this signal into efficient later evaluation and control implementation.

The emphasis on latency is also consistent with the intermediate position of P2 and N2 in the processing stream. Because these components occur between early sensory registration and later controlled evaluation, they may be especially vulnerable to slowed processing and state instability without necessarily showing a uniform amplitude reduction. ERP latency measures can reveal delayed onset or slower accumulation of control-related processing even when peak magnitude remains stable [135]. In cognitive control paradigms, N2 is also shaped by expectancy and proactive control settings, meaning that its timing and amplitude depend partly on whether control is already engaged before the imperative stimulus [155]. Under sleep deprivation, proactive control is likely to be less stable, so delayed N2 may reflect slower engagement of preconfigured control states or a shift toward more reactive, stimulus-driven control.

The working memory literature provides a further explanation for the heterogeneity of P2/N2 effects. In context-updating and working memory paradigms, P2-range activity has been linked to early context or template updating and target evaluation, whereas N2 has been linked to mismatch discrimination between current input and maintained representations [145,156]. Visual and n-back studies also show that P2 and N2 are sensitive to stimulus structure, cognitive load, and even preprocessing choices, which may contribute to variability across sleep deprivation paradigms [157,158]. Thus, inconsistent P2/N2 amplitude findings do not necessarily indicate contradictory evidence. Instead, they likely reflect a combination of genuine mechanistic differences, including compensation versus inefficiency, and the inherent dependence of these components on task design and analytic decisions.

### 4.5. Performance Monitoring After Sleep Deprivation: ERN/Ne–Pe Dissociation

The error-related findings are best interpreted within the broader ERP literature on performance monitoring, which distinguishes between early error detection and later conscious-evaluative processing. ERN/Ne and Pe are usually treated as partially dissociable stages of a response-monitoring cascade: ERN/Ne is an early frontocentral negativity associated with rapid action monitoring or error detection, whereas Pe is a later centroparietal positivity more closely related to conscious error evaluation, error awareness, motivational salience, and downstream behavioural adaptation [159,160]. This distinction provides a clear framework for the reviewed findings: sleep deprivation appears to disrupt later evaluative processing of errors more consistently than the earliest error-detection signal.

Mechanistically, this ERN–Pe dissociation is compatible with the main models of performance monitoring. Conflict-monitoring accounts link ERN to medial frontal detection of post-response conflict, whereas reinforcement-learning accounts interpret ERN as a teaching signal generated when outcomes are worse than expected [161,162,163]. Although these models differ in emphasis, both place ERN generation within fast medial–frontal monitoring circuitry that can operate before full conscious appraisal of the error. This helps explain why ERN can remain relatively preserved in some sleep deprivation paradigms, while Pe is reduced more reliably: early monitoring may still detect that something has gone wrong, but the later stage that integrates this signal into conscious evaluation and adaptive control is more vulnerable to fatigue.

The error awareness literature strongly supports this interpretation. Classic antisaccade and error awareness studies showed that errors can elicit a substantial ERN even when participants are unaware of them, whereas Pe is much larger for consciously perceived errors and reduced or absent for unperceived errors; unperceived errors also tend to lack normal post-error slowing [164,165,166]. Applied to sleep deprivation, attenuated Pe with preserved or only modestly reduced ERN suggests not a complete collapse of performance monitoring, but a failure of the error signal to reach, stabilise, or engage later conscious–evaluative systems. In other words, the sleep-deprived brain may still generate a rapid internal alarm but may be less able to transform that alarm into awareness, certainty, and adaptive behavioural adjustment.

This interpretation is further supported by evidence that conscious error awareness depends on the accumulation and integration of multiple post-response evidence sources, including motor, sensory, interoceptive, and contextual signals [166,167,168]. Sleep deprivation is likely to impair this later integration stage by increasing lapses, uncertainty, and state instability. Thus, reduced Pe may reflect weakened evidentiary buildup after an error rather than absence of the initial monitoring signal. This also explains why reduced Pe often coincides with poorer post-error adjustment, even when mean accuracy or reaction time changes are modest.

The functional similarity between Pe and other late positivities is also important. Pe has often been described as “P3-like,” reflecting context updating or salience evaluation in response to having made an error rather than a passive consequence of ERN generation [166,169,170]. This places Pe reduction within the broader sleep deprivation pattern observed across the P3 family, LPC/P600, LPP, and other late resource-dependent components. The common mechanism is likely impaired late-stage allocation of evaluative resources, reduced integration of task-relevant evidence, and weaker updating of behaviourally significant events.

By contrast, the heterogeneity of ERN findings is not surprising. Contemporary EEG accounts treat ERN and related monitoring signals as part of broader medial–frontal control dynamics, especially frontal-midline theta activity associated with conflict, error processing, and the need for cognitive control [171,172]. Sleep deprivation may therefore alter ERN in several ways: by reducing error sensitivity, increasing neural noise, disrupting theta-based coordination, or changing the relative contribution of conflict, salience, and adaptation processes within the response-locked time window [171,172]. Because ERN is also sensitive to motivation, anxiety, task engagement, and control strategy, baseline individual differences and experimental context may buffer or amplify its vulnerability to sleep loss [159,162,163,166,171].

Motivational manipulations fit this account. ERN amplitude is known to increase when errors are more motivationally significant or when performance is strongly emphasised, whereas Pe is more closely tied to later subjective evaluation and awareness [159,162,166]. Therefore, incentives or high task engagement may preserve early error reactivity under sleep deprivation, but still fail to restore Pe if fatigue disrupts evidence accumulation, salience integration, or conscious access. Put simply, motivation may keep the early alarm system responsive without fully restoring the later evaluative stage.

### 4.6. Novelty Detection, Orienting, Selective Attention, and Reorienting

The novelty- and attention-related findings are best interpreted within a hierarchical ERP model in which deviance detection, orienting, selective enhancement, and reorienting are partially separable stages of processing. In this framework, MMN indexes automatic deviance detection, P3a or novelty P3 reflects involuntary orienting toward unexpected or potentially relevant events, and RON reflects reorienting back to the primary task after distraction [127,173,174]. This organisation is useful for interpreting sleep deprivation effects because it shows that attentional disruption does not have to occur uniformly across the whole processing chain. Sleep loss may spare early deviance detection while weakening later orienting, selective enhancement, or task re-engagement.

The common pattern of preserved MMN with reduced P3a is especially informative. MMN is generally understood as an automatic comparison between incoming input and a short-term regularity model, and it can be generated without active task engagement. Because this process is relatively early and pre-attentive, it may remain relatively resistant to fatigue. By contrast, P3a is more strongly associated with salience-driven orienting, interruption of ongoing processing, and recruitment of frontal attention control systems [127,174,175]. Therefore, reduced P3a after sleep deprivation suggests that the brain may still detect deviance but becomes less able to orient efficiently towards that deviance and evaluate its behavioural relevance.

This distinction is important for interpreting behavioural distraction effects. A reduced P3a, or reduced behavioural distraction by novel stimuli, should not be interpreted as improved attentional control. In the broader distraction literature, P3a reflects the orienting response to potentially relevant novelty, not simply a failure to suppress distraction. Therefore, weaker P3a after sleep deprivation may indicate blunted responsiveness to novelty rather than superior filtering. In this sense, reduced distraction costs can coexist with poorer cognitive integrity because the system is less effectively registering the significance of unexpected events [173].

The frontal novelty-P3 findings further support this interpretation. Novelty P3/P3a has long been associated with frontal attention-control mechanisms, while lesion, source-localisation, and multimodal studies support a distinction between frontal P3a and more parietal P3b activity [127,176,177]. Thus, reduced or delayed frontal novelty P3 after sleep deprivation, especially when accompanied by a more posterior scalp distribution, is best interpreted as weakened frontal novelty evaluation with possible compensatory redistribution toward posterior associative processing. In this case, topographic change is not merely a descriptive finding; it suggests altered weighting within the networks that evaluate novelty and allocate attention.

Later positivity and reorienting effects indicate that novelty processing may also be displaced downstream under sleep deprivation. Novelty-related processing is not limited to the P3a peak. Later positivities and reorienting-related negativities can reflect continued evaluation, control recovery, or task re-engagement, particularly when frontal orienting is inefficient or when distracting events impose additional control demands [178,179,180]. Therefore, altered early novelty responses after sleep loss may not simply disappear; in some paradigms, they may shift processing demands toward later recovery or re-engagement stages.

Selective attention findings can be explained using the same stage-specific logic. Classic auditory ERP studies show that selective attention enhances early auditory responses, especially N1, and produces a longer-lasting negative difference wave often termed processing negativity or Nd, reflecting selective enhancement of task-relevant input [181,182]. Importantly, attended–unattended differences are often driven more by enhancement of attended input than by suppression of unattended input. Accordingly, sleep-deprivation-related reductions in N1 or Nd attention effects are best interpreted as weakened top-down enhancement of relevant sensory streams rather than as a simple increase in distractor processing.

This interpretation is strengthened by network-level evidence showing that N1 enhancement and longer-latency processing negativity arise from distributed auditory, temporal, and frontoparietal systems [183,184]. Sleep deprivation may therefore leave basic auditory registration relatively intact while impairing the control signals that bias early encoding toward task-relevant input. This fits the broader pattern of ERP findings reviewed here: sleep loss does not necessarily abolish early responses, but it reduces their selectivity, stability, and dependence on task goals.

RON findings are particularly important because they identify a later stage of attentional control that is distinct from both deviance detection and initial orienting. In the involuntary-attention literature, RON is usually interpreted as reflecting reorienting back to the main task after distraction and is placed downstream of MMN and P3a, although coupling between these components is not obligatory across paradigms [173,174,185]. Therefore, preserved MMN and preserved or only mildly altered P3a can coexist with abnormal RON if sleep deprivation primarily disrupts re-engagement with task goals rather than deviance detection or initial orienting. In this sense, RON abnormalities support a mechanism of impaired attentional recovery after distraction.

A broader implication is that sleep deprivation effects on attention-related ERPs should be expected to vary across paradigms. MMN, P3a, Nd, and RON index different computations: sensory comparison, orienting, selective enhancement, and reorienting. These stages need not be affected in parallel. MMN may remain stable while P3a declines; P3a may remain detectable while RON changes; and N1/Nd selective-attention effects may weaken independently of novelty responses [127,173,186]. This heterogeneity is therefore not necessarily contradictory. Rather, it reflects the fact that different tasks stress different points in the attentional chain.

Task context and working memory load likely determine where sleep deprivation effects become most visible. Distraction-related ERP responses are shaped by available control resources, and working memory demands can alter both orienting and reorienting responses [127,174,186,187]. Because prolonged wakefulness reduces effective control capacity, the same novel or distracting event may still trigger MMN but produce reduced P3a, altered RON, or weakened selective-attention effects depending on how many resources remain available for orienting, task reconfiguration, and goal re-engagement. Thus, the most defensible interpretation is not that sleep deprivation produces a single global attentional deficit, but that it selectively weakens the transitions from automatic detection to orienting, selective enhancement, and recovery of task focus.

### 4.7. CNV and Anticipatory Preparation: Impaired Readiness Before Stimulus Onset

The CNV findings suggest that sleep deprivation disrupts anticipatory preparation before stimulus onset, not only stimulus-evoked processing after a target appears. Since its original description, CNV has been interpreted as a slow preparatory potential developing between warning and imperative signals, reflecting expectancy, attentional set, and motor readiness rather than a single isolated process [188,189,190,191]. Within this framework, reduced CNV amplitude or area after sleep deprivation indicates impaired maintenance of preparatory readiness, whereas delayed CNV subcomponents suggest slower build-up of expectancy and action preparation. Occasional CNV increases are also interpretable within the same framework, but they should be viewed cautiously as possible compensatory over-recruitment rather than evidence of improved readiness [188,189,190,191].

This interpretation is supported by ERP-fMRI evidence linking CNV generation to distributed preparatory networks, including thalamic, supplementary motor, anterior cingulate, insular, somatomotor, and frontostriatal regions [191,192]. CNV attenuation under sleep deprivation can therefore be understood as impaired coordination within an anticipatory-control network rather than as a nonspecific fatigue effect. Prolonged wakefulness may weaken the ability of thalamic gating, medial–frontal control, and motor preparatory systems to maintain an optimal state before the imperative stimulus. As a result, the sleep-deprived brain may still process the target when it appears, but it does so from a less prepared and less stable baseline.

The functional relevance of CNV is further supported by studies showing that larger or better-maintained CNV activity is associated with faster responses [191]. This helps explain why CNV deterioration and reaction-time slowing often co-occur under sleep deprivation. Both likely reflect reduced efficiency of preparatory networks. In this sense, slowed behaviour after sleep loss should not be attributed only to delayed post-stimulus evaluation or motor execution; it may also arise because the system fails to establish adequate readiness before the stimulus occurs.

A key point is that CNV is not a unitary component. Classical and contemporary accounts distinguish earlier CNV activity, more closely related to orienting and expectancy, from later terminal CNV activity, more closely linked to motor preparation and controlled attentional effort [188,189,193]. This distinction helps explain why sleep deprivation studies report reductions, delays, or mixed amplitude effects depending on how CNV is measured. Reduced CNV area, delayed subcomponents, and attenuated terminal CNV should not be treated as contradictory findings. Instead, they may reflect impairment at different points in a multistage preparatory process: the initiation of expectancy, the maintenance of the preparatory set, the build-up of motor readiness, or the conversion of temporal prediction into action preparation.

Frontal system findings provide an additional mechanistic explanation for why CNV is sensitive to sleep loss. Late CNV is preferentially reduced after dorsolateral prefrontal damage, whereas early CNV can remain relatively preserved; reduced late CNV is also associated with slower responses and poorer preparatory differentiation [191,193]. By analogy, sleep deprivation may produce a transient functional downregulation of lateral and medial frontal preparatory systems. The circuitry remains structurally intact, but prolonged wakefulness reduces its capacity to maintain readiness across time. This fits the broader pattern observed in this review, in which later and control-dependent ERP components are more vulnerable than early sensory responses.

The CNV literature also indicates that anticipatory preparation is not purely motoric. CNV reflects time-based preparation built on temporal expectations, but it should not be interpreted as a direct measure of interval timing itself [190]. Slow preparatory activity can also emerge in passive paradigms without explicit response demands when participants learn temporal contingencies between events [189]. Therefore, CNV abnormalities after sleep deprivation may reflect disruption at multiple anticipatory levels: weaker temporal expectancy, poorer maintenance of attentional set, and impaired translation of prediction into motor readiness. Sleep-deprived individuals may still detect temporal regularities, but they appear less able to use them to prepare efficiently for upcoming events.

Paradoxical CNV increases under some sleep deprivation conditions can be explained by compensatory arousal and effort. CNV amplitude is known to vary with expectancy, motivation, preparatory effort, and catecholaminergic activation [188,190,191]. Thus, when participants can still recruit compensatory control resources, CNV may transiently increase as the system works harder to maintain readiness. However, with deeper or more prolonged sleep loss, compensatory capacity may fail, producing the more typical pattern of reduced CNV amplitude, reduced CNV area, delayed timing, and slower responses.

Finally, CNV appears closely linked to arousal regulation. CNV-related activity in cingulate, supplementary motor, and insular regions has been associated with peripheral autonomic arousal, suggesting that CNV reflects how preparatory circuits are tuned by current physiological state [192]. This is especially relevant to sleep deprivation, which destabilises arousal and wake maintenance. Under sleep loss, CNV may therefore index the moment-to-moment ability to convert arousal into sustained preparatory cortical activation. This may also explain why CNV abnormalities can persist after partial recovery sleep in some protocols: subjective alertness and gross performance may improve before the coupling between arousal regulation and preparatory control is fully restored.

### 4.8. Semantic Processing, Recognition Memory, and Sleep-Dependent Representational Updating

The semantic and recognition-memory findings extend this stage-specific pattern to meaning-related and mnemonic processing. In the broader ERP literature, the N400 is one of the most reliable markers of meaning-related processing. Its amplitude is strongly modulated by contextual fit, semantic relatedness, and expectancy, whereas its latency is comparatively stable. Current accounts therefore treat the N400 as reflecting context-sensitive semantic access, integration, or prediction error rather than isolated lexical activation [194,195]. This framework helps explain why preserved semantic differentiation can coexist with reduced overall N400 amplitude after sleep deprivation: basic semantic structure may remain accessible, while the mechanisms that sustain expectancy, contextual constraint, or proactive semantic preparation become weaker.

This interpretation is supported by semantic-priming ERP studies showing that N400 effects include both automatic and controlled contributions depending on stimulus timing and task demands [196,197,198]. In paradigms with longer stimulus onset asynchronies or stronger strategic demands, participants rely more heavily on expectancy generation and post-lexical checking. These controlled semantic operations are especially likely to be vulnerable to sleep loss. Accordingly, reduced prolonged anterior negativity and altered N400-family responses after deprivation are best interpreted as evidence of impaired maintenance of predictive semantic set, rather than as a complete breakdown of semantic access.

The divergence between picture–word and word–word priming effects can be understood in the same way. N400 effects depend strongly on stimulus format, perceptual encoding demands, and whether the task encourages proactive prediction or reactive integration [194,196,199]. A picture–word task may reveal sleep-sensitive changes at earlier stages because picture encoding imposes greater perceptual and semantic mapping demands. By contrast, a long-SOA word–word task is more likely to expose deficits in strategic expectancy and controlled semantic preparation. Thus, sleep deprivation appears to reweight the processes supporting semantic performance rather than producing a uniform impairment across all forms of semantic processing.

Recognition-memory findings show a similar stage-specific pattern. The ERP literature on old/new effects distinguishes an earlier mid-frontal old/new effect, often labelled FN400, from a later parietal positivity or LPC [200,201,202,203]. Although the functional meaning of the FN400 remains debated, the LPC is more consistently associated with recollective retrieval, post-retrieval evaluation, and decision-relevant mnemonic elaboration [204]. This distinction helps explain why earlier and mid-latency old/new effects may weaken after accounting for vigilance-sensitive variance, whereas the LPC/P600 reduction remains more robust. Sleep deprivation may leave some early item-related or familiarity-like signals partially available, while impairing the later amplification and evaluation required for controlled recollection.

The use of vigilance-sensitive covariation is important for this interpretation. ERP methodology reviews emphasise that early and mid-latency old/new effects can be influenced by nonspecific state factors such as arousal, attention, and perceptual fluency, whereas later effects are more diagnostically linked to retrieval operations when these factors are controlled [201,205,206]. Therefore, when an LPC/P600 deficit remains after controlling for vigilance-related activity, it is more plausibly interpreted as a genuine impairment in controlled mnemonic evaluation rather than merely a consequence of reduced alertness. In practical terms, the memory trace may still be activated, but the sleep-deprived brain is less able to elaborate, stabilise, and use it for recollective discrimination.

The consolidation findings extend this account beyond immediate fatigue effects. ERP studies of word learning and semantic association learning show that N400 attenuation across exposure or after offline intervals is a marker of strengthened semantic links and easier meaning integration [207,208]. N400 and LPC measures are therefore useful for tracking the transition from fragile episodic traces toward more integrated lexical–semantic representations, even when behavioural changes are modest [111,207,208]. Against this background, the absence of normal overnight N400 attenuation after wakefulness suggests that sleep deprivation interferes with representational strengthening itself, not only with alertness at test. Although accompanying N1 attenuation supports a dual state-plus-memory interpretation, the missing N400 attenuation is especially informative because changes in N400 over time are more closely tied to semantic integration than to transient response strategy [90,209].

There is also a conceptual link between these language and memory findings and the broader pattern of late ERP vulnerability observed elsewhere in this review. Late positivities such as LPC and P600-family responses often reflect controlled integration, reanalysis, decision relevance, or conscious evaluation, and some accounts propose partial functional overlap with domain-general P3b-like updating processes [196,210]. Without reducing LPC, P600, and P3b to the same component, this literature supports a broader mechanism: sleep deprivation disproportionately disrupts the late, resource-dependent processes that transform an activated representation into a stable, reportable, and task-relevant decision or integration outcome.

### 4.9. Affective and Social Processing: Altered Emotional Salience, Regulation, and Face-Related ERPs

The affective and social ERP findings are best interpreted within a multistage model in which emotional information is processed through partly dissociable stages of perceptual encoding, motivational salience allocation, conflict monitoring, regulatory control, and sustained evaluation. This framework is important because sleep deprivation does not appear to produce a uniform impairment of emotional processing. Instead, its effects vary depending on whether the task primarily engages early face perception, threat detection, emotional conflict adaptation, empathy-related processing, or sustained regulation of emotional salience.

The late positive potential (LPP) provides one of the clearest examples of sleep-loss-related disruption in affective processing. In the broader ERP literature, the LPP is widely understood as a sustained positivity associated with emotional significance and motivated attention, while remaining sensitive to top-down regulation strategies such as reappraisal and attentional redirection [211,212,213]. Therefore, the loss of normal LPP down-regulation after sleep deprivation is best interpreted as impaired neural implementation of emotion regulation rather than merely as a change in subjective emotional experience. Participants may still report some degree of regulatory success, but the ERP evidence suggests that sleep loss reduces the capacity to modulate sustained motivated attention at the neural level [213,214,215].

The passive-viewing findings can be explained using the same LPP framework. Reduced emotional–neutral differentiation, particularly when driven by increased LPP responses to neutral stimuli, does not necessarily indicate emotional blunting. Because LPP reflects stimulus significance and motivated attention rather than emotional valence alone, increased neutral-stimulus LPP after sleep deprivation may reflect reduced selectivity of salience assignment or poorer filtering of motivational relevance [210,212,213]. In this sense, sleep loss may cause neutral stimuli to receive abnormally sustained processing, thereby weakening the contrast between emotional and neutral categories.

The emotional conflict findings also support a compensatory rather than beneficial interpretation of increased late positivity. Face–word emotional Stroop studies show that emotional conflict engages a sequence of conflict-sensitive negativities and later parietal positivities, with the latter linked to conflict resolution, post-response monitoring, and adaptation [216,217,218]. Therefore, increased P3-like activity in specific conflict-adaptation contexts after sleep deprivation should not be interpreted as improved emotional control. A more plausible interpretation is that the sleep-deprived brain recruits additional late evaluative resources to maintain performance when conflict processing becomes less efficient.

Early face-processing effects show that sleep deprivation can also alter perceptual-emotional tuning. The N170 is no longer considered strictly insensitive to facial expression; meta-analytic evidence indicates that it is modulated by emotional content, especially anger and fear [219]. Thus, emotion- and ambiguity-dependent P1 or N170 changes after sleep deprivation are consistent with the broader face-ERP literature. They suggest that sleep loss can reshape early perceptual prioritisation of socially relevant cues, particularly when stimuli are ambiguous or threat-related.

This interpretation is strengthened by evidence that early face-emotion responses depend on task goals, contextual relevance, and observer state. Studies using task manipulations and repetition paradigms show early N170 sensitivity to fear even when emotion is not task-relevant, while broader reviews indicate that facial-expression ERPs are substantially influenced by contextual information [220,221,222]. Therefore, the reviewed sleep deprivation findings should not be interpreted as a general impairment of face processing. Rather, prolonged wakefulness appears to alter which facial and emotional cues receive early perceptual priority, especially when threat salience or ambiguity increases.

The empathy-for-pain findings require a more cautious interpretation because early empathy-related ERP effects are methodologically heterogeneous. Earlier ERP work proposed a two-stage model in which relatively early frontal and central N1/N2 responses reflect more automatic affective sharing, whereas later P3/LPP responses reflect more controlled evaluative processing. However, more recent meta-analytic evidence suggests that early N1/N2 empathy effects are less reliable and more variable than later central-parietal P3/LPP effects [223,224]. Against this background, reduced N2/N340 with preserved LPP in a sleep deprivation paradigm may indicate disruption of earlier perceptual-attentional or resonance-like processing while leaving later sustained evaluation relatively intact. However, because early empathy-related components are variable across paradigms, this conclusion should be treated as task-specific rather than generalised too broadly.

A broader point emerging from this section is that subjective reports and neural evidence may diverge after sleep deprivation. The emotion-regulation ERP literature shows that self-reported affect and LPP modulation are related but not interchangeable, and can dissociate depending on strategy use, age, psychopathology, and regulatory success [213,214,215]. This is directly relevant to sleep deprivation studies: participants may report successful regulation or limited emotional change, while ERP measures reveal reduced neural selectivity, altered salience assignment, or impaired top-down modulation. ERPs are therefore particularly useful in this domain because they help identify which stage of affective or social processing is disrupted when behavioural or self-report measures remain ambiguous.

### 4.10. Sensory Gating, Salience Processing, and Modality-Specific ERP Effects

The sensory-gating and modality-specific findings suggest that sleep deprivation alters the stability, timing, and selectivity of sensory processing rather than abolishing sensory registration. Across auditory, brainstem, and nociceptive paradigms, the reviewed studies showed a combination of delayed latencies, weakened filtering, preserved but reorganised early responses, and variable amplitude changes. This pattern is most consistent with state-dependent disruption of sensory gain, prediction, gating, habituation, and salience assignment rather than a simple reduction in sensory responsiveness.

The auditory findings are particularly informative because they show that preserved early responses can coexist with impaired sensory regulation. In the broader auditory ERP literature, N1 is strongly shaped by stimulus history, presentation rate, feature-specific adaptation, and cortical excitability [225,226,227,228]. Therefore, findings such as preserved N1 refractoriness with altered N1 amplitude should not be treated as contradictory. They suggest that local adaptation mechanisms may remain partly intact, while the overall responsiveness of auditory cortex shifts under sleep loss. Depending on arousal state, task context, and baseline excitability, this shift may produce either increased or decreased amplitudes rather than a uniform direction of change [225,226,227,228].

The P50 findings provide a clearer example of impaired early filtering. In paired-click paradigms, P50 suppression is usually interpreted as a rapid sensory-gating mechanism that limits the cortical response to redundant input, whereas N100 and P200 gating reflect partly distinct and somewhat later filtering or attentional operations [227,228,229]. Therefore, reduced P50 suppression after sleep deprivation, especially when the response to the first stimulus remains preserved, is best interpreted as weakened inhibitory filtering rather than sensory failure. Prolonged wakefulness appears to reduce the brain’s ability to protect downstream processing systems from redundant sensory input, thereby increasing noise and placing greater demands on later attention and control mechanisms.

Brainstem and cortical latency findings further indicate that sleep deprivation can affect sensory processing at multiple levels. The ABR/BAEP literature treats Wave I and Wave V latencies, as well as inter-wave intervals, as markers of transmission timing along the ascending auditory pathway [230,231]. Thus, delayed BAEP latencies together with delayed cortical MMN or P300 responses suggest multilevel slowing of auditory processing rather than a purely cortical executive delay. In other words, sleep deprivation may perturb the timing of the auditory processing chain from early transmission through cortical comparison and later stimulus evaluation.

The nociceptive findings require a similarly nuanced interpretation. Laser-evoked potentials are not simple readouts of pain intensity. The broader LEP literature shows that the vertex N2–P2 complex is strongly influenced by salience, attentional capture, contextual predictability, and repetition, whereas the earlier N1 is more closely related to lateralised sensory-nociceptive processing [232,233,234]. Therefore, reduced average P2 after sleep deprivation does not necessarily imply reduced nociceptive processing, particularly when subjective pain increases. A more plausible interpretation is that sleep loss alters salience allocation and habituation dynamics across repeated painful stimuli, changing the averaged waveform while pain experience may still intensify.

Habituation is especially important for interpreting these pain-related ERP effects. N2–P2 habituation to repeated noxious stimulation is a robust phenomenon, but its magnitude depends on timing, expectation, context, and individual factors, and it can dissociate from subjective pain ratings [232,235]. The reviewed findings therefore suggest that sleep deprivation may modify the rate or form of cortical adaptation to repeated noxious input rather than simply increasing or decreasing pain-evoked activity. Mechanistically, prolonged wakefulness may disturb the balance between salience-driven orienting to painful stimuli and adaptive down-weighting of repeated input, producing the apparently paradoxical combination of greater pain experience with faster or altered ERP habituation.

A common mechanism across the auditory and nociceptive findings is disrupted salience calibration. Novelty-P3/P3a and LEP studies indicate that late vertex-positive responses are closely related to orienting and evaluation of important sensory events, not only to modality-specific encoding [127,232,233,234]. In the nociceptive domain, several accounts argue that much of the vertex LEP complex reflects a multimodal salience detection system rather than pain-specific activity alone [232,233,234]. From this perspective, sleep deprivation does not eliminate responses to sensory events, but makes the system that prioritises, filters, and adapts to those events less stable and less efficiently calibrated.

This interpretation also explains why amplitude effects are variable across sensory modalities. ERP amplitude reflects a mixture of sensory drive, adaptation, attention, expectancy, salience, and current arousal state. Sensory-gating work shows that different components have different relationships to attention and executive performance, and LEP studies show that N2/P2 magnitude can vary with salience and repetition independently of perceived pain [227,229,232,233]. Therefore, larger responses in some auditory contexts, smaller responses in nociceptive paradigms, or preserved amplitudes with delayed latencies should not be interpreted as inconsistent findings. They are better understood as different expressions of state-dependent reweighting of sensory gain, filtering, prediction, and salience processing.

### 4.11. Oscillatory State, Phase Locking, and Temporal Instability of ERP Responses

The oscillatory and phase-locking findings suggest that sleep deprivation can degrade ERP responses not only by reducing the strength of component-specific neural activity, but also by increasing the temporal instability of event-related processing. Conventional ERP averages depend on the repeated alignment of neural responses across trials. Therefore, when sleep deprivation increases trial-to-trial latency jitter or reduces phase consistency, averaged ERP waveforms may become smaller, broader, or less reliable even if the underlying response is still generated on many individual trials. Methodological and simulation studies show that ERP amplitude, latency, temporal jitter, and inter-trial phase coherence are closely interrelated, meaning that apparent amplitude reductions under sleep deprivation may partly reflect impaired temporal reproducibility rather than simple loss of neural responsiveness [133,236,237].

This interpretation is supported by the broader literature on pre-stimulus oscillatory state. Alpha power and phase, together with other ongoing oscillatory features, influence the magnitude and timing of early visual ERPs and perceptual performance [238,239,240,241]. Thus, findings linking pre-stimulus theta, alpha, and beta activity to later ERP attenuation under sleep deprivation fit a general principle of ERP generation: the brain’s response to a stimulus depends partly on the neural state in which that stimulus arrives. Sleep deprivation likely amplifies this dependence because tonic arousal becomes more unstable. As a result, stimuli are more likely to be processed during low-excitability, poorly phased, or otherwise suboptimal neural states, producing weaker, delayed, or more variable phasic responses.

A state-instability account is therefore more informative than a simple amplitude-loss account. Pre-stimulus alpha and related oscillatory measures are not merely passive correlates of alertness; they are linked to cortical excitability, sensory gating, and the efficiency of feedforward processing [242,243,244]. Under sleep deprivation, rising sleep pressure and fluctuating arousal may increase variability in these gating states. Consequently, ERP responses become more dependent on momentary pre-stimulus conditions rather than being uniformly reduced across all trials. This mechanism helps explain why sleep-deprived participants may show intermittent lapses, variable reaction times, and inconsistent ERP amplitudes despite still producing recognisable component waveforms.

The SSVEP and vigilance findings further support this view. In the broader SSVEP and ITPC literature, inter-trial phase coherence indexes the temporal consistency of stimulus-locked responses and attentional engagement. SSVEP amplitude and ITPC can dissociate, suggesting that they capture partly different aspects of neural processing, with ITPC being especially sensitive to the precision of entrainment across trials [245,246]. Reduced ITPC or phase-locking value under sleep deprivation therefore suggests that prolonged wakefulness impairs the brain’s ability to repeatedly align neural activity to external events or rhythms. This should be understood as a deficit in timing stability and entrainment reliability, not merely as weaker evoked amplitude.

At the same time, phase-locking metrics require cautious interpretation. Methodological work shows that ITPC changes are not independent of ERP amplitude, latency, waveform sharpness, or signal-to-noise properties [133,236,247,248]. This is particularly important in sleep deprivation studies because amplitude reductions and latency delays often occur together. Therefore, reduced phase locking under sleep loss should not be interpreted as a pure phase-resetting deficit in isolation. The safest interpretation is that it reflects reduced temporal reproducibility of event-related processing, which should be evaluated together with amplitude, latency, and behavioural variability.

This interpretation also fits broader models of ERP generation. ERPs are increasingly understood as arising from a combination of additive evoked responses, stimulus-driven phase alignment, and changes in the power of ongoing oscillations, rather than from a single mechanism alone [249,250,251,252]. Sleep deprivation may therefore degrade ERP morphology through several converging pathways: weaker evoked recruitment, poorer phase alignment, noisier baseline oscillatory activity, and greater trial-to-trial variability in neural readiness. In this sense, smaller or less sharply defined ERP components after sleep loss may reflect impaired coordination of distributed neural activity rather than reduced activity in one isolated component generator.

Oscillatory instability also links naturally to cognitive control findings. Frontal-midline theta has been proposed as a mechanism for coordinating control across distributed cortical regions, particularly during conflict monitoring, error processing, and adaptive regulation [171,253]. Sleep deprivation often increases tonic theta activity as a marker of rising sleep pressure, but this background increase does not necessarily imply more efficient control. Instead, elevated tonic theta and unstable oscillatory dynamics may interfere with the precise timing needed for phasic control responses. This provides a plausible bridge between oscillatory state changes and the observed vulnerability of N2, ERN, P3, Pe, and other control-related ERP components.

Finally, temporal instability helps explain why ERP findings after sleep deprivation can vary across studies without requiring fundamentally different mechanisms for each component. Increased latency jitter can make averaged peaks appear attenuated; stronger pre-stimulus alpha or theta fluctuations can produce a mixture of normal and impaired trials; and reduced phase locking can make components appear noisier or less reliable even when their generators remain partly active. Thus, state instability should not be treated as an additional finding separate from amplitude and latency changes. Rather, it may be one of the hidden mechanisms that produces them in averaged ERP waveforms [133,237].

### 4.12. Recovery, Naps, Countermeasures, and Component-Specific Restoration

The next pattern is also strongly supported by the broader ERP literature and is best interpreted as a component-specific restoration problem rather than a binary impaired-versus-recovered state. Different ERP components index partially separable processes, such as preparatory readiness, conflict monitoring, stimulus evaluation, and late conscious evaluation, and they differ in both reliability and state sensitivity. This matters for recovery studies because a short intervention can normalise one stage of processing while leaving another abnormal. Test–retest work confirms that many cognitive ERPs are stable enough to support longitudinal interpretation, but also shows that reliability differs across components and metrics, with some latency measures often more stable than amplitudes [254,255,256].

This reliability background helps explain why the reviewed studies repeatedly find partial recovery in one ERP metric but not others. In the broader methods literature, amplitude and latency are not interchangeable: latency is more directly linked to processing speed and temporal dynamics, whereas amplitude is more sensitive to resource allocation, state-dependent factors, and trial-to-trial variability. Patterns such as improved latency alongside persistent amplitude abnormality, or the reverse, are therefore mechanistically plausible and should be expected in recovery paradigms. Recovery should be defined at the level of specific components and metrics rather than inferred from a single ERP summary measure [254,255,256].

The discussion of naps and immediate post-nap effects is likewise consistent with the broader nap and sleep inertia ERP literature. Classic P300 studies show that post-nap recovery follows a time-dependent trajectory: latency may be prolonged immediately after awakening because of sleep inertia, then shorten as inertia dissipates and alertness improves [257,258]. This provides a strong mechanistic model for the reviewed nap results: naps can restore processing speed relatively quickly, but the earliest post-awakening window may still be impaired, especially on latency-based measures.

The same literature also supports the idea that nap benefits can extend across multiple processing stages. In non-sleep-deprivation paradigms, P300 latency is often used as a marker of stimulus-evaluation speed, whereas CNV indexes expectancy and preparatory readiness. Because these processes rely on partly overlapping but distinct networks, simultaneous improvement in P300 and CNV after a nap is best interpreted as coordinated restoration of both post-stimulus evaluation and pre-stimulus readiness. The broader CNV literature consistently describes CNV as a frontocentral preparatory potential linked to expectancy, cortical alertness, and motor readiness, supporting the interpretation that nap-related CNV changes reflect genuine recovery of readiness rather than incidental waveform shifts [257,258,259].

The emphasis on component-specific pharmacologic rescue is also well grounded in the ERP pharmacology literature. Modafinil studies outside the sleep deprivation ERP literature report shortened N2 and P300 latencies together with improved information processing speed and attentional orientation, consistent with a broad enhancement of processing efficiency rather than a narrow sensory effect [260]. This directly supports the interpretation that modafinil can rescue both intermediate and later stages, including N2, P3, and in some reviewed studies CNV-related timing, by improving overall efficiency and reducing the need for compensatory over-recruitment.

By contrast, the caffeine ERP literature supports a more selective and stage-limited rescue profile, which aligns closely with the reviewed findings. Experimental ERP studies of caffeine in Go/NoGo and related paradigms show robust modulation of earlier and mid-latency components, especially P2/N2-family effects, whereas effects on later P3 components are often weaker, less consistent, or more context-dependent [112,261,262,263]. This provides an important mechanistic parallel to the review: caffeine may improve engagement and inhibitory selection processes without fully restoring later evaluative allocation—a distinction that behavioural measures alone often miss.

Findings on recovery and countermeasures also align with the broader literature distinguishing motivation-sensitive from evaluation-sensitive performance monitoring. Non-sleep ERP research shows that ERN and Pe are dissociable and differentially modulated by motivation, affect, and task demands. ERN appears particularly sensitive to motivational and aversive significance, whereas Pe is more closely tied to later conscious evaluation and adaptive adjustment [264,265,266,267]. This offers a plausible explanation for findings that incentives can preserve early error reactivity without restoring Pe or later adaptive control: motivation may maintain early monitoring salience but does not necessarily reinstate downstream evaluative processing.

Similar logic applies to training and resilience effects, such as meditation-related attenuation of ERP disruption in the reviewed studies. The broader ERP literature increasingly treats components such as CNV, N2, and P3 as markers of modifiable control systems rather than fixed traits. CNV studies in particular suggest that amplitude and latency parameters reflect expectancy and readiness in ways that are sensitive to both state and intervention [259,268,269]. This makes training-based resilience entirely plausible: such interventions may not normalise cognition globally, but they can alter the vulnerability of preparatory and evaluative systems, which then appears as component-specific changes in CNV and P3 trajectories under challenge.

Finally, the idea that ERP recovery may precede behavioural recovery is well supported by general ERP theory and longitudinal work. ERPs can detect covert changes in processing speed, resource allocation, or monitoring before those changes are large enough to alter aggregate accuracy or mean reaction time. This is partly because behavioural outputs compress multiple processing stages into a single measure, whereas ERP components sample the processing stream at multiple points. Reliability studies further indicate that these component-level measures can be stable and sensitive enough to track subtle recovery trajectories even when overt behaviour appears unchanged [254,255,256]. This directly supports the interpretation of nap and intervention studies in which N2, P3, or CNV improve before performance catches up.

## 5. Homeostatic vs. Circadian Influences: Two-Process Model Integration

The neurocognitive impairments can be understood in the context of Borbély’s two-process model of sleep regulation, which describes sleep–wake dynamics as the interaction of two processes: a homeostatic drive (Process S) that increases with time awake and dissipates with sleep, and a circadian drive (Process C) governed by the internal clock that promotes wakefulness at certain times of day. Cognitive alertness and performance at any moment are essentially the result of these two processes combined—one reflecting how long one has been awake, and the other reflecting the time in the 24 h biological cycle. Sleep deprivation places these processes in conflict: Process S grows inexorably, raising the pressure for sleep and impairing neurobehavioural functioning, while Process C may temporarily counteract this pressure during the biological daytime but eventually wanes at night. ERP findings in sleep-deprived individuals strongly reflect this interplay. In Borbély’s model, total sleep propensity is the sum of S (homeostatic sleep pressure) and C (circadian signal), and high sleep propensity (especially when driven by elevated S) corresponds to reduced vigilance and slowed cognition. Empirical studies have indeed tied rising homeostatic sleep pressure to progressive slowing of brain responses: for instance, as hours of wakefulness accumulate from morning into the night and beyond, the P300 latency keeps lengthening in a roughly linear fashion. One experiment that kept participants awake ~36 h found P300 latency increased significantly from ~296 ms at baseline to ~332 ms at 36 h awake, and similarly the CNV late component was ~11–12% slower by 24–36 h awake. These changes track the buildup of Process S, illustrating the homeostatic effect of extended wakefulness on cognitive processing speed. In the same study, allowing the subjects to recover with sleep brought these ERP latencies back closer to baseline, consistent with a restoration of low Process S after recovery sleep [270].

Importantly, circadian timing modulates the severity of cognitive deficits at a given level of sleep deprivation. Under normal conditions, the circadian Process C promotes wakefulness during the day and into the evening, which helps maintain alertness even as one has been awake for many hours (moderate Process S). For example, when participants are well rested, P300 latencies actually tend to speed up slightly in the late evening compared to morning—presumably because the circadian drive for alertness is high in the evening, counteracting fatigue. However, under sleep-deprived conditions this usual circadian pattern is altered. One study observed that after a night without sleep, P300 latency no longer showed the normal evening improvement; instead, by the late evening of the deprivation day the P300 was markedly slower (∼57 ms longer) compared to the same time of day when rested. In other words, with process S at extreme levels, the brain could not capitalise on the circadian boost it would ordinarily get in the evening. In fact, the high homeostatic load led to disproportionately sluggish cognition at times when performance would normally be relatively good. This underscores that circadian misalignment or being awake during one’s biological night exacerbates cognitive deficits. When the circadian pacemaker is at its low ebb (e.g., in the pre-dawn hours) and Process S is high, the two processes align to produce maximal sleep propensity, resulting in very poor vigilance and profound ERP slowing. Consistent with this, some attention-related brain measures show anomalous “bottoming out” around the circadian trough: for instance, the amplitude of the CNV (which requires sustained effort) can show unexpected lapses or variability around dawn, a time when the circadian drive for wakefulness is minimal. Within the framework of the two-process model, the early morning hours are when Process C can no longer offset the mounting Process S, leading to a collapse in alertness. The model has been shown to account for performance deterioration during circadian misalignment experiments, such as forced desynchrony protocols, through the interaction of a single circadian oscillator (Process C) with the homeostatic process (Process S). Alexander Borbély and colleagues demonstrated that a wide range of complex alertness patterns during prolonged wake could be simulated by a two-process interaction without invoking multiple pacemakers, highlighting that cognitive vigilance is fundamentally joint-governed by sleep homeostasis and circadian phase [270].

The two-process model also illuminates phenomena observed during recovery sleep and the aftermath of sleep deprivation. Rebound sleep following deprivation is characterised by unusually high intensity (e.g., elevated slow-wave activity), reflecting the dissipation of a greatly accumulated Process S. Behaviourally, recovery sleep tends to restore cognitive performance substantially, but not always completely, depending on the duration and timing of recovery. After one full night of recovery sleep, many behavioural metrics (e.g., psychomotor vigilance and go/no-go accuracy) return to baseline or near-baseline levels, indicating that the acute homeostatic drive has been relieved. ERP measures show partial normalisation as well. For example, after 36 h awake, 8 h of recovery sleep brought P300 latencies and CNV latencies back down to baseline levels in one study, and P300 amplitude, which had shown aberrations during deprivation, reverted towards normal. Similarly, in a 36 h total sleep deprivation study with a go/no-go task, a night of recovery eliminated the significant difference in go-task accuracy and produced a clear rebound in the NoGo P3 amplitude compared to the sleep-deprived state. Nevertheless, ERP indices sometimes reveal residual deficits even when behaviour appears recovered. In the go/no-go study, although one night of recovery sleep restored Go performance to baseline, the NoGo P3 and the frontal N2 still did not fully return to well-rested amplitudes. The authors interpreted this incomplete electrophysiological recovery as evidence of lingering neurocognitive effects, that the brain’s deeper executive networks had not entirely normalised despite the outward performance improvements. This aligns with the two-process model in that a single sleep period may not erase all homeostatic pressure if the deprivation was severe; some “sleep debt” can remain, manifesting in subtle cognitive and EEG changes. It is also consistent with the notion of local sleep and differential recovery: after prolonged waking, certain cortical circuits (especially prefrontal networks underlying executive ERPs) might need more than one night to fully recover their baseline functional capacity, even though overall alertness (a more global measure) rebounds more quickly [270].

Another important aspect of sleep/wake regulation is sleep inertia—the transient grogginess and cognitive impairment upon awakening from sleep, particularly from deep sleep or at suboptimal circadian times. Sleep inertia can be viewed as the immediate aftereffects of high Process S and low Process C upon awakening. ERP studies have provided insight here as well. When individuals are awakened abruptly from sleep (for example, during the biological night or after a recovery sleep rich in slow-wave sleep), early sensory–perceptual ERPs are significantly blunted and slowed for several minutes. In one experiment, the amplitude of the auditory N1–P2 complex was dramatically reduced immediately after awakening compared to before sleep, and this effect was especially pronounced after a recovery night that had an elevated slow-wave sleep rebound. Additionally, the N1 latency was found to be prolonged after awakening from sleep (by tens of milliseconds) relative to pre-sleep baseline, indicating a slowing of even early auditory processing during the first moments of wakefulness. These effects were not as strong when people were awakened from a normal baseline night, but were very large when they were awakened from a recovery night with high homeostatic pressure (lots of Stage N3 sleep). This provides direct evidence that high Process S at the moment of awakening impairs cortical responsiveness, consistent with the concept of sleep inertia. The two-process model explains this as the result of the brain being temporally still in a “sleep mode”—Process S remains elevated locally and the circadian drive may still be low (if awakening occurs in the early morning), so the brain cannot instantly switch to an optimal awake state. In practical terms, this is why cognitive performance is often very poor immediately after waking from a nap or a deep sleep: the ERPs show reduced amplitude and slowed latency, mirroring the subjective and behavioural sluggishness that gradually dissipates as Process S continues to drop and circadian drive rises after awakening [270].

It should be noted that the timing of recovery sleep relative to circadian phase can also influence the pattern of recovery, as the two-process model predicts. If recovery sleep or naps occur at an unusual biological time, the interplay of S and C may produce a two-stage recovery. Borbély observed in early studies that when animals were allowed recovery sleep beginning in their normal active phase (circadian misalignment), they showed a biphasic rebound in sleep intensity rather than a smooth return to baseline. By analogy, humans taking a recovery nap at an odd circadian time might initially restore only part of their performance (Process S is reduced but Process C might counteract sleep depth), and full restoration may not occur until the principal sleep period resumes at the proper circadian night. The model thus emphasises that optimal cognitive recovery requires both homeostatic repayment and circadian alignment [270].

## 6. Conclusions

This systematic review of 82 human studies indicates that sleep deprivation produces a reliable but non-uniform disruption of ERPs, with the most consistent effects observed in mid-to-late cognitive components rather than early sensory responses. Across paradigms, the P300/P3 family emerges as the clearest electrophysiological signature of sleep loss, typically showing latency prolongation and/or amplitude reduction, consistent with slower stimulus evaluation, reduced attentional resource allocation, and impaired context updating under prolonged wakefulness. In some tasks, larger P3 responses appear instead, but these are best interpreted as temporary compensatory recruitment rather than preserved function.

A central cross-study conclusion is that sleep deprivation affects information processing in a stage-specific manner. Early sensory and pre-attentive components (e.g., P1, N1, MMN, P50) are often relatively preserved, but this preservation is selective: sleep loss can still slow their timing, weaken gating/filtering, reduce phase consistency, or impair attention-dependent modulation. By contrast, later components indexing inhibitory control, conflict monitoring, performance monitoring, memory evaluation, emotional regulation. and sustained attentional control are more consistently altered, supporting the view that prolonged wakefulness primarily degrades stability, selectivity, and control of processing, not simply basic sensory registration.

The review also highlights that ERP abnormalities can be present even when behavioural outcomes are modest, variable, or apparently preserved, underscoring the value of ERPs as sensitive mechanistic markers of covert neural inefficiency and compensation. In particular, findings on reduced phase locking and increased state dependence indicate that sleep deprivation impairs not only component amplitude and latency, but also the temporal precision and trial-to-trial stability of neural processing. This state-instability account helps explain why performance under sleep deprivation is often inconsistent and lapse-prone.

Recovery findings further indicate that the effects of sleep loss are not reversed uniformly. Naps, recovery sleep, and countermeasures (e.g., modafinil, caffeine, motivational manipulations, training-related resilience) yield partial and component-specific restoration. Certain ERP indices normalise earlier than others and, in some cases, precede observable behavioural recovery. This pattern underscores a key methodological and theoretical point: “recovery” should be defined at the level of specific ERP components and metrics (amplitude vs. latency), rather than as a binary recovered/not recovered state.

At the same time, the evidence base has notable limitations. The literature is dominated by small, highly selected samples of healthy young adults, often with male overrepresentation, substantial attrition due to EEG artifact rejection or prolonged wakefulness protocols, and considerable heterogeneity in deprivation protocols, ERP paradigms, preprocessing pipelines, and outcome definitions. These factors limit generalisability and preclude quantitative meta-analysis in the present review.

Overall, the reviewed evidence supports a coherent conclusion: sleep deprivation primarily disrupts higher-order, late-stage, and temporally coordinated neural processing. Earlier sensory processing is often retained but becomes slower, less stable, and less selectively regulated. ERPs are therefore well suited to mapping the stage-specific effects of sleep loss and identifying neural systems that are vulnerable, compensatory, or differentially responsive to recovery. Future work should prioritise larger and more diverse samples, tighter methodological standardisation, transparent reporting of preprocessing and measurement choices, and designs that better dissociate homeostatic and circadian influences, to support translation of ERP markers into clinical and operational fatigue monitoring.

## Figures and Tables

**Table 1:** Studies included in the review.

Recovery/Nap/Other Modifiers	Main ERP Effects	Behavioural/Subjective Effects	Task/ERP Focus	Sleep Manipulation	Sample	Study
30 min nap mitigated cognitive slowing and ERP latency changes	TSD: P300 latency ↑; CNV M1 latency ↑. Nap: P300 latency and CNV M1 latency improved toward baseline	Sleepiness ↑; RT slowed after TSD; nap improved sleepiness and RT	Auditory oddball (P300); CNV task	24 h TSD; +30 min nap (1–3 a.m.) condition	9 healthy young men	[34]
Zaleplon: short-term cost, later ERP timing benefit	TSD and abrupt awakening: NoGo-P3 latency ↑, amplitude ↓. Zaleplon caused worse immediate NoGo-P3 latency (sleep inertia), but better later (4–6 h) latency vs. placebo	Inhibition performance worsened after TSD; nap helped over time; no strong behavioural zaleplon-placebo difference	Visual Go/NoGo (NoGo-N2, NoGo-P3)	30 h TSD; 2 h nap + zaleplon vs. placebo	16 male undergrads (crossover)	[35]
TSD mainly affected later cognitive processing, not selective peripheral vision	No “tunnel vision” ERP signature. P300 amplitude ↓ (latency unchanged). Early sensory ERPs mostly unchanged. Parvocellular P100 latency ↑ (specific effect)	RT slower and omissions ↑ globally (central + peripheral equally)	Tunnel-vision visual task; pattern-reversal VEP/ERP	~27 h TSD vs. rested	19 professional truck drivers	[36]
Shows selective vulnerability of vigilance/simple speed	P300 latency ↑, amplitude ↓ (both amplitude metrics); no major time-of-day interaction	Vigilance and simple RT worsened; complex cognitrone performance relatively preserved (practice effects noted)	Auditory oddball P300 + neurocognitive tests	38 h TSD (4 repeated sessions)	30 college students	[37]
Brief recovery sleep did not produce durable P300 recovery; later decline persisted	No reliable latency effects. Peak-to-peak P300 amplitude declined by 6 h post-recovery (Cz/Pz significant)	Not primary emphasis	Auditory oddball P300 at Fz/Cz/Pz	~24 h TSD + 110 min recovery sleep; tested 30 min, 3 h, 6 h later	14 analysed (17 enrolled) students	[38]
P300/N200 changes tracked sleepiness; P200 amplitude tracked mood/anxiety/fatigue	P300 latency ↑, amplitude ↓; N200 latency ↑, amplitude reduced; N100 unchanged; P200 amplitude ↑	Sleepiness, fatigue, anxiety, negative mood ↑	Auditory AERP (N100, P200, N200, P300) + mood/fatigue scales	37 h TSD (morning/evening repeated sessions)	24 college students	[39]
Portable EEG system detected TSD effects mainly in P300 amplitude	P300 amplitude decreased in TSD group (group × time); N100 unchanged; N400 time effect but interaction not significant; latencies largely unchanged	Behavioural data collected but reported separately	NeuroCatch “brain vital signs” (N100, P300, N400)	Overnight TSD vs. control	30 healthy adults (15 TSD, 15 control)	[40]
TSD changed normal evening “speeding” pattern of P300 latency	P300 latency ↑ (~27 ms overall); amplitude unchanged; N100 unaffected. Circadian pattern of P300 latency altered by TSD	Sleepiness markedly ↑; RTs showed time-of-day effects; P300 latency correlated with RT only when sleep deprived	Auditory oddball P300 (Pz focus)	One-night TSD vs. normal sleep; 3 sessions/day	18 students (15 ERP datasets)	[41]
Nightshift history moderated RT impact more than ERP latency impact	P300 latency ↑ (~18.8 ms overall); amplitude ↓ trend (ns)	RT slowed overall; acute group slowed much more than chronic nightshift group	Auditory oddball P300 + RT	24 h TSD	26 adults (13 acute/no nightshift, 13 chronic nightshift)	[42]
TSD selectively weakened voluntary (endogenous) attentional selection at early stage	Early P1 unaffected by day. Parietal N1 reduction for endogenous spatial cues only (not exogenous). P2 ↑, P3 ↓ generally with TSD	RT slowed; accuracy dropped modestly; timeouts increased sharply	Exogenous vs. endogenous ANT; visual ERPs (P1, N1, P2, P3)	24 h TSD	26 military personnel (14 cadets, 12 soldiers)	[43]
8 h recovery sleep partially restored behaviour and ERPs, not fully	TSD caused latency prolongation + amplitude reductions across P200/N200/P300 (frontal prominence); P200 highlighted as “information replacement” marker	Accuracy ↓, misses ↑, RT slower under TSD; partial improvement after recovery	2-back pronunciation WM; P200/N200/P300	Baseline (TIB) → 36 h TSD → 8 h recovery sleep (TIBR)	31 postgraduate men	[44]
Supports dissociation: top-down/sustained attention impaired, more automatic control relatively spared	Go N200 amplitude ↓/latency delayed; Stop-P3 amplitude ↓; Pe amplitude ↓. Stop-N200 and ERN largely preserved	Go accuracy (sustained attention) dropped strongly; SSRT/stopping accuracy largely unchanged	Stop-signal task; go/stop N200/P300, ERN, Pe	Baseline + maximally rested + 24 h TSD	24 healthy young adults	[45]
TSD impairs WM; P2 increase interpreted as possible compensation	P3 amplitude ↓, P2 amplitude ↑; N2 and P2 latencies ↑; P3 latency ↑ trend (ns)	Accuracy ↓ and throughput ↓; RT trend slower (ns)	2-back WM (pronunciation/spatial/object); P2/N2/P3	Baseline vs. 36 h TSD	14 analysed (16 recruited) male students	[46]
Evidence for parietal compensation under TSD	P2 preserved (no TSD main effect). P3 ↓ in frontal/central but ↑ in parietal (compensatory redistribution)	Accuracy ↓ in both tasks	Visual WM updating (spatial/object 2-back); P2/P3	Baseline vs. 36 h TSD	14 healthy young men	[47]
TSD disrupted hemispheric lateralisation (right-hemisphere advantage)	P3 amplitude ↓, N2 latency ↑; P3 latency ↑ trend (ns). Right-hemisphere P3 advantage present at baseline, lost after TSD	Accuracy ↓ and throughput ↓; RT trend slower (ns)	Spatial 2-back WM; lateralised N2/P3	Baseline vs. 36 h TSD	14 analysed (16 recruited) men	[48]
TSD effects are more evident at higher WM load, especially in P3	N2 unaffected. P3 latency ↑ (TSD + load effects); P3 amplitude showed TSD × load interaction (largest reduction in 2-back)	RT/accuracy mainly load effects; efficiency metric showed TSD × load interaction	1-back vs. 2-back pronunciation WM; N2/P3	Baseline vs. 36 h TSD	22 men (ERP *n* = 20)	[49]
TSD effects were age-dependent and concentrated on alerting/orienting (N1) in young	TSD selective: no P1 or P3 latency effect; auditory N1 latency prolonged in young only. Aging affected many ERP stages (early faster, later P3 slower/smaller)	Focus on multitask driving performance context	Driving + spatial visual/auditory discrimination; visual P1/N1/P3, auditory N1/P2/P3	Normal sleep vs. one night TSD	41 male drivers (young vs. old)	[50]
Strong evidence of impaired error monitoring (ERN) and inhibitory control under TSD	Flanker P300 delayed, Go-P300 amplitude ↓, NoGo-N2 ↓, ERN reduced (latency unchanged). Pe differences nuanced (larger in poor-performing TSD subgroup)	Slower responding; lower Go/NoGo accuracy; more NoGo false alarms	Flanker + Go/NoGo; P300, NoGo-N2, ERN, Pe	Acute overnight TSD vs. rested	49 young adults (TSD vs. rested control)	[51]
Broad slowing across processing stages, with executive-control ERP weakening	All component latencies prolonged (N1/P2/N2/P3). N1 amp unchanged; P2 amp ↑; NoGo-N2 and NoGo-P3 amplitudes ↓, NoGo-P3 latency ↑	RT slower; omissions and commissions ↑ in TSD	Visual Go/NoGo; N1/P2/N2/P3	~43 h TSD vs. no-sleep-deprivation control	24 male undergrads (TSD vs. NSD)	[52]
Behavioural recovery > neural recovery after one recovery night	NoGo-P3 amplitude decreased dose-dependently with TSD, partial rebound after recovery. NoGo-N2 latency ↑ with TSD, partial recovery; N2 amp relatively preserved	Go hit rate declined after 24/36 h; recovered after sleep. RT and false alarms relatively stable	Visual Go/NoGo; NoGo-N2/NoGo-P3	36 h TSD + 8 h recovery sleep (5 time points)	12 valid ERP datasets (14 recruited) men	[53]
Isolates TSD-specific effect beyond isolation stress	P300 amplitude showed Time × Condition interaction: post-test Go-P300 much smaller after TSD vs. isolation. N200 no comparable TSD-specific effect	Positive affect ↓, mood disturbance ↑ under TSD vs. SI	Numeric Go/NoGo; N200/P300 + mood + autonomic	72 h TSD vs. 72 h social isolation (with sleep)	11 analysed men	[54]
Suggests broad deficit in stimulus evaluation/attention, not only inhibition	P3 amplitude declined ~50% across wakefulness for both rare Go and rare NoGo; not inhibition-specific. Recovery improved but not fully	Vigilance ↓, false alarms ↑ (NoGo), Go RT slowed later in deprivation	Auditory Go/NoGo with equal rarity for critical Go/NoGo; P3	36 h TSD with repeated assessments + recovery sleep	11 analysed students	[55]
Athletic training may buffer some early conflict/error-monitoring ERP effects	P3 amplitude ↓ in both groups. Controls showed N2 and ERN reductions after TSD; athletes showed little/no N2 or ERN change (possible resilience).	SSRT worsened in both groups; athletes remained faster. Stop accuracy drop clearer in controls	Stop-signal task; N2, P3, ERN	Baseline vs. 36 h TSD	28 men (15 controls, 13 table-tennis athletes)	[56]
8 h recovery sleep improved but only partially normalised WM ERPs/performance	TSD: N2 latency ↑, P3 amplitude ↓ (“low-amplitude, slow-wave” pattern). After recovery: N2 latency improved, P3 amplitude restored upward	Accuracy and throughput ↓ after TSD; improved after recovery; RT not robust	3 WM 2-back tasks; N2/P3	36 h TSD + 8 h recovery sleep; control sleep group	42 men (NS *n* = 20; SD *n* = 22; ERP slightly fewer)	[57]
Strong evidence for frontal novelty-processing vulnerability to TSD	Novel P3 frontal amplitude ↓ and latency ↑ after TSD; late parietal novelty positivity ↑. Target P3 amplitude ↓ and latency ↑. Recovery normalised ERP pattern	More misses/false alarms and RT variability after TSD; recovery largely normalised performance	Novelty oddball: novel P3 and target P3	36 h TSD + recovery night	24 students (12 TSD, 12 control)	[58]
Sleep loss weakens attention capture (P3a) while preserving early deviance detection (MMN)	MMN preserved (early deviance detection intact). P3a reduced after TSD and PSD (weaker involuntary attention capture). P3b reduced esp. after TSD	Overall task performance worsened with sleep loss, but deviant “distraction cost” was reduced under TSD	Auditory duration task with irrelevant pitch deviant; MMN/P3a/P3b	Total SD and partial SD (within-subject)	Exp1 *n* = 11 (TSD), Exp2 *n* = 14 (partial SD)	[59]
TSD increased sleep pressure and favored sleep-protective responses rather than arousal-like dampening	No flattening: N350/N550/P900 tended to be larger post-TSD (esp. louder tones); P220/P450 no deprivation effect	Sleep-onset latency drastically shorter post-TSD; fewer arousals/spindles, more VSWs/K-complexes	Sleep-onset/NREM auditory ERPs (P220, N350, P450, N550, P900) + K-complex/spindles/arousals	One night TSD; pre/post deprivation bedtime nap protocol	10 analysed (12 enrolled) students	[60]
Parallel behavioural and ERP attention decline after TSD	P300 latency ↑ (~61 ms), amplitude ↓	Selective attention worsened (RT, omissions, stability); sustained tracking worsened	Auditory Go/NoGo ERP (P300 at Cz) + selective/sustained attention tests	~24–32 h TSD	19 young adults	[61]
Meditation training appeared to ameliorate many TSD-related cognitive/ERP disruptions	Pre-meditation: P300 latency ↑ at 36 h, CNV latencies/RT worsened; multiple amplitudes changed. Post-meditation: many deprivation-related ERP/CNV changes reduced/absent	Raven reasoning worsened with TSD pre-intervention; improved after recovery; less deterioration post-meditation	Auditory P300, CNV, MLR + Raven	Pre–post 60 days meditation; TSD at 24 h & 36 h + recovery	10 Indian Army men	[62]
48 h recovery restored measures toward baseline. Actigraphy used to verify no naps.	TSD ↑ N100 and P300 latency (P300 paradigm), ↑ CNV-related M100/P300 latencies; amplitudes mostly unchanged (P200/P300, CNV amps). Modafinil reduced/normalised latency slowing.	TSD ↑ sleepiness (SSS/ESS), ↑ counting errors, ↑ RT; modafinil prevented sleepiness rise, improved errors/RT toward baseline.	Auditory oddball P300 + CNV (warning–imperative RT task)	24 h TSD; repeated-measures across baseline, post-TSD, 48 h recovery; second phase repeated with modafinil 400 mg/24 h during TSD	11 healthy young men (20–35)	[63]
Ecological shift-work model; order counterbalanced; 7-day sleep diary quantified sleep.	No major early visual cortex loss; for targets, sleep loss shifted early source activity toward prefrontal (BA9/10, BA8/9) in P1/N1 windows (compensatory recruitment). No significant P3 effect.	↑ SSS, worse mood (BDI), poorer immediate recall; sustained-attention accuracy largely preserved, only limited early RT slowing.	Continuous Attention Test + ERP source localisation (LORETA); P1/N1/P3 time windows	Real-world on-call partial sleep loss vs. night at home (within-subject; ~3.9 h vs. 7.5 h sleep)	16 male physicians (mean 29.6)	[64]
Suggests TSD shifts from effortful expectancy strategy to more automatic semantic activation.	Word task: N400 priming pattern preserved, but overall N400 smaller after TSD; prime-to-target anterior slow negativity attenuated (reduced expectancy/preparatory activity). P200 unchanged. Picture task: no N400 sleep effect; early prime-picture negativity increased after TSD (70–130 ms).	Strong ↑ sleepiness after TSD; behaviour mostly preserved in word task; in picture task, accuracy drops mainly for weak/unassociated pairs.	Word–word and picture–word semantic priming; ERPs: P200, N400, late positivity, plus prime-related slow negativity	One night TSD vs. normal sleep (counterbalanced, ≥1 week apart)	12 healthy adults (6F/6M), ages 20–31 (ERP analyses smaller after exclusions)	[65]
Structured recovery sleep showed broad ERP normalisation except higher-order P718 timing.	Early sensory ERPs (0–132 ms) largely intact. From ~140 ms onward: widespread amplitude reductions (esp. N382, P718), latency slowing for several components; P718 latency shortened. Most recovered after sleep except P718 latency remained abnormal.	RT slowed progressively; ERP amplitude/latency changes correlated with RT slowing. Prestimulus theta/alpha/beta power increased and inversely related to ERP amplitudes.	Visual vigilance/discrimination; visual ERPs decomposed by PCA + prestimulus EEG power	40 h TSD, repeated testing every 2 h (21 sessions), then recovery sleep in three 3 h blocks	8 healthy men (22–30)	[66]
Highlights nonlinear trajectory with 24 h critical point and partial late compensation (24–36 h).	Significant time effects for P2, N2, P3 amplitudes. Major drop by 24 h; P2/N2 show partial rebound by 36 h (compensatory), while P3 remains depressed.	Accuracy declines mainly 12→24 h then plateaus; RT not significant.	Spatial 2-back; ERPs: P2 (150–200), N2 (200–300), P3 (300–500)	Repeated-measures at 0 h, 12 h, 24 h, 36 h continuous wakefulness	20 students recruited; 18 analysed (14M/6F initially)	[67]
ERN/Pe reductions remained even in accuracy-matched subsample (not just performance artifact).	TSD reduced ERN and Pe substantially; N2 not reduced (slightly larger/delayed, possible compensation).	Slower RT, more variability, lower accuracy, more errors/omissions. Post-error remedial adjustment lost after TSD; post-conflict adaptation preserved.	Modified flanker; ERPs: N2, P300, ERN, Pe + spectral power	Within-subject: normal sleep vs. one night TSD (~25.5 h awake at testing)	16 healthy young adults (7 women)	[68]
Combined task-ERP + resting-network approach; suggests network disconnection underlies inhibition deficits.	NoGo-N2 more negative and delayed (compensatory/conflict monitoring), NoGo-P3 smaller and delayed (impaired later inhibition). High-alpha resting FC decreased (esp. DMN/visual links); FC changes correlated with N2/P3 changes.	Slower Go RT, lower Go hit rate, higher NoGo false alarms (poorer inhibition).	Go/NoGo task ERPs (NoGo-N2, NoGo-P3) + resting EEG alpha-band functional connectivity (PLV/NBS)	Within-subject baseline vs. 36 h TSD	25 healthy young men	[69]
Female-only sample; emotional inhibition did not show amplified TSD cost relative to shape task.	Resting theta increased (~33%); alpha unchanged. TSD reduced N2 go–no-go difference (both tasks). P3 go–no-go difference reduced in shape task only (not emotional). N170 go/no-go difference unchanged.	Lower GNG accuracy, slower RT; emotional task harder overall but no larger TSD × emotion interaction. KINARM task: more distractor hits (↑ distractibility) after TSD.	Resting EEG + shape and emotional face Go/NoGo; ERPs at Cz: N2, P3, and N170 (emotional task)	Within-subject randomised: normal sleep vs. overnight TSD	12 healthy females (18–27)	[70]
Connectivity changes correlated with accuracy changes (not RT) in identical condition.	P300 amplitude decreased after TSD (identical & mirror trials). Source activity reduced in parietal/precuneus/frontal-temporal areas. Connectivity reconfigured (↑ some left frontal→parietal links, ↓ others).	Performance worsened mainly for identical trials (↑ RT, ↑ errors); angle effects preserved.	Letter mental rotation; ERP P300 (300–500 ms) + eLORETA + directed effective connectivity (iCoh)	One-group pre/post: baseline vs. 36 h TSD	30 men recruited; 24 analysed	[71]
Sequence-dependent (conflict adaptation) effects are key; compensatory P300 despite worse behaviour.	P300 amplitude altered by TSD, with sequence-sensitive 3-way interaction; notably iC trials showed more positive P300 in SD (compensatory attentional allocation).	TSD ↑ sleepiness, PVT lapses/slowing, lower positive affect. In task: more errors, slower RT; specific vulnerability after congruent-previous trials.	Emotional conflict (face-word Stroop) with sequence effects (cC/cI/iC/iI); ERP P300 at CPz	Within-subject randomised/counterbalanced: normal sleep vs. 24 h TSD	25 healthy young adults (18–30)	[72]
Incentives partially buffered ERN and trended to shorten P300 latency under TSD, but no global protection.	TSD ↓ P300 amplitude, ↓ Pe, ↑ ERN latency; ERN amplitude showed incentive × sleep interaction: preserved with incentives but dropped without incentives. FRN (in incentive group) unaffected by sleep.	TSD increased sleepiness, RT/RT variability, reduced accuracy, impaired post-error accuracy adjustment. Incentives improved accuracy/motivation but did not broadly rescue TSD deficits.	Letter flanker; ERPs: P300, ERN, Pe, FRN	Mixed design: incentives (between) × normal sleep vs. one night TSD (within)	24 university students (12 incentive/12 no-incentive; ERP *n* = 20)	[73]
Separate control repetition group showed no comparable LPP flattening (rules out mere retest effect).	After TSD, LPP no longer differentiated emotional vs. neutral effectively; main driver was increased LPP to neutral pictures (flattened emotional–neutral contrast).	Negative pictures rated less negative after TSD (emotional blunting); no major RT-rating latency effects. Anxiety increased after picture viewing at baseline but not after TSD; cortisol unchanged.	IAPS picture viewing + ratings; ERP LPP (300–800 ms)	Within-group baseline morning vs. post-overnight TSD morning; control group repeated sessions without TSD	TSD experiment: 12 recruited, 10 analysed; separate no-SD control group *n* = 10	[74]
Authors used N100 covariate to separate vigilance from memory-specific ERP changes.	TSD reduced vigilance-linked N100. LPC/P600 old/new effect reduced after TSD (memory-specific). LFC and some N400 session effects appeared partly vigilance-dependent (covaried with N100). Also reduced left-posterior N200 modulation (vigilance-independent).	Trend toward worse correct rejection of new faces; slower false-alarm RTs after TSD (discrimination/monitoring difficulty).	Face recognition old/new; ERPs N200, P250, N400, LPC/P600, late frontal component (LFC)	Within-subject: Sleep vs. TSD between study evening and test next morning	18 healthy students (9F/9M)	[75]
Shows selective vulnerability of ACC-like early monitoring, not broad behavioural collapse.	Ne/ERN amplitude reduced after TSD, especially for corrected errors and specifically in stimulus-incongruent trials; Pe not significantly changed.	Overall performance/correction metrics mostly unchanged, but immediate correction rate selectively reduced in stimulus-incongruent trials under TSD.	Modified flanker with immediate correction response; response-locked ERPs Ne/ERN, Pe	Within-subject counterbalanced: normal sleep vs. one night TSD	16 healthy undergrads (7F/9M)	[76]
Mixed evidence for impairment; task/hazard type dominated effects more than sleep.	Sleep effect mainly interactional: N1 latency longer in SD only for no-hazard scenes; N1 amp unchanged. N2 amplitude varied by hazard type (largest for overt hazards) but not by SD.	Hazard type strongly affected performance (covert hazards hardest/slower). No broad SD RT effect; accuracy interaction showed SD group more accurate on covert hazards.	Hazard perception images (no/covert/overt hazard); ERPs N1 (100–150), N2 (250–350)	Between-subject: overnight SD vs. normal sleep (verified with actigraphy/SSS)	56 novice drivers analysed (28 SD, 28 control)	[77]
Key dissociation: SD affects earlier visual stage (N1), alcohol affects later stage (N2).	SD selectively delayed Oz N1 latency. Alcohol selectively delayed Cz N2 latency (esp. ~0.08 BAC) in alcohol-only condition; N2 effect not significant when combined with SD. P3 visually suppressed by alcohol (not formal main analysis).	RT slowed with alcohol and SD in prior/parallel findings; N2 latency correlated with RT during treatment sessions.	Simple visual RT with EMG-defined RT; visual ERPs P1, N1, P2, N2, P3	Counterbalanced within-subject sleep vs. 30 h SD across lab stays; alcohol dose between-subject; ERPs recorded post-dose	54 male volunteers (some excluded from ERP analyses); randomised to placebo/low/moderate alcohol dose	[78]
Social isolation control showed no comparable ERP change; sleep loss effect specific to feedback-stage neural processing.	Behavioural IGT net score changes not significant. After TSD, N250–400 amplitude reduced post vs. pre (esp. frontal/central tendency), indicating weakened feedback evaluation.	No reliable net-score deterioration detected (small sample/low power likely).	IGT with feedback-locked ERP; N250–400 (FRN-like) at Fz/Cz/Pz	Within-subject comparison including 72 h TSD and social isolation control (counterbalanced)	12 male students; 11 analysed post-dropout	[79]
Trial-count matched analyses confirmed effects not due to fewer artifact-free trials in TSD.	In regular sleep: robust attended > unattended negativity in both windows. In TSD: attention modulation abolished/reduced (no reliable attended–unattended effect). Later processing negativity deficit driven mainly by reduced response to attended probes (reduced signal enhancement).	Story comprehension remained high (slight trend lower in TSD).	Dichotic listening with/ba/probes; ERPs N1 (150–250) and processing negativity (300–450) to attended vs. unattended probes	Between-subject: regular sleep vs. 24 h TSD (overnight monitored)	35 analysed (20 regular sleep, 15 TSD; between-subject)	[80]
Highlights preserved early change detection but altered reorienting stage under TSD, modulated by age.	MMN unchanged by TSD/age. P3a smaller in older adults (age effect), no reliable sleep effect. RON showed age × sleep interaction (older adults’ larger RON reduced after TSD), age-related latency slowing persisted.	TSD reduced standard-trial accuracy advantage; RT distraction cost attenuated in young under TSD; residual post-deviant slowing increased under TSD (esp. trend in older adults).	Auditory duration discrimination with irrelevant frequency deviants; deviant-minus-standard ERPs MMN, P3a, RON	Within-subject: normal sleep vs. 26 h TSD; age group between-subject	20 adults: 11 young, 9 older	[81]
Baseline PSG-controlled design; points to selective sadness-processing vulnerability plus altered threat-related compensation.	General: SD → smaller P1 and larger/more negative N170 (FF). MF task: N170 showed group × emotion × morph interaction—under SD, increasing N170 for subtle threat (fear/anger) but decreasing N170 for sad as ambiguity increased.	SD slowed RT overall and impaired accuracy especially for sad faces (FF and MF); altered error biases (more “happy” responses to negative faces, less “sad”).	Full-face and morphed-face emotion categorisation; ERPs P1, N170	Between-subject after baseline PSG night: second night normal sleep vs. overnight SD; test at ~14:30 next day	49 healthy adults (control vs. SD; ERP Ns smaller after artifact exclusions)	[82]
Exploratory clinical study (no Bonferroni); medication heterogeneity present.	Whole group: N2 and P300 latencies prolonged at frontal sites; P300 amplitude reduced at several sites. Responder/nonresponder differences strongest in N1: responders had smaller baseline N1 amplitudes and showed post-TSD N1 amplitude increases; nonresponders showed N1 latency decreases and P300 amplitude decreases.	Clinical response defined by HDRS improvement (≥30%); ERP study aimed at physiological correlates of response.	Auditory oddball; ERPs N1, P2, N2, P300; responder vs. nonresponder analyses	~40 h TSD (one total night + following day), pre/post ERP at 10 a.m.	17 depressed female inpatients (9 responders, 8 nonresponders)	[83]
Train design emphasised short-lived within-sequence trace formation; suggests preserved preattentive change detection under this paradigm.	N1 refractoriness pattern preserved (Sleep × Position ns), but overall N1 amplitude larger after TSD. MMN not significantly changed by TSD.	SSS markedly higher after TSD.	Passive auditory train oddball while reading; N1 refractoriness across repeated standards + MMN (5th standard vs. deviant)	Within-subject: control (morning/evening) and post-TSD (morning/evening), conditions ~2 weeks apart	22 healthy undergrads recruited; 20 ERP analysed	[84]
Controlled time-isolation lab; no naps/microsleeps allowed.	Robust MMN remained but MMN amplitude decreased at 24 h and 36 h (strongest for larger 10% pitch deviants). N1 not reduced (sometimes more negative), implying intact sensory encoding but weaker deviance comparison.	Pre-attentive change detection subtly deteriorated with prolonged wakefulness.	Passive auditory oddball (pitch deviants while doing visual game); ERP MMN and N1	36 h TSD, ERP at baseline, 24 h, 36 h	14 healthy male students	[85]
Moderate/realistic sleepiness (not full TSD); baseline chosen to avoid pre-response P300 contamination.	Ne/ERN unchanged (core amplitude ns). Pe significantly reduced in sleepy condition.	Participants felt sleepier and rated performance worse, but error counts and error estimates similar. Post-error slowing reduced when sleepy (less behavioural adjustment).	Flanker task; response-locked ERPs Ne/ERN and Pe	Within-subject: alert (~4 h awake) vs. sleepy (~20 h awake; ~3 h after bedtime)	17 healthy women (19–45)	[86]
Key contrast: chronic restriction impaired awareness/Pe more than acute TSD in this paradigm.	TSD (Exp1): no significant changes in ERN or Pe. Sleep restriction (Exp2): ERN preserved, but Pe reduced with condition × session × awareness effects.	TSD: sleepiness ↑, but no reliable changes in error rate, error awareness probability, or awareness RT. SR: sleepiness ↑ cumulatively; reduced error awareness and slower awareness RT, especially for repeat NoGo as restriction accumulated.	Error Awareness Task (Go/NoGo + awareness button); ERPs ERN, Pe (trial-level models)	Exp1: ~35 h TSD, tested well-rested and ~27 h awake. Exp2: chronic SR (3 nights of 3 h TIB) vs. well-rested (4 nights 9 h TIB), repeated sessions	Exp1 TSD: 14 healthy adults; Exp2 SR: 27 adults (EEG subset *n* = 21)	[87]
Nonlinear “dip then rebound” pattern suggests compensatory adaptation around 36 h for awareness/Pe.	ERN stable across stages. Pe reduced at SD-24 vs. baseline, then rebounded by SD-36 (not different from baseline); Pe positively correlated with error-awareness rate.	Fatigue/mood worsened at SD-24 and SD-36. PES present at all stages but smaller during SD. PIA absent/worse at SD-24, then emerged at SD-36. Error awareness rate/RT worst at SD-24, improved by SD-36.	Arrow flanker with explicit correctness judgment; ERPs ERN, Pe	Within-subject repeated measures at pre-SD, SD-24, SD-36	33 healthy students (16M/17F)	[88]
Strong dissociation: background low-frequency power rises while stimulus-locked precision (P1/PLI) falls.	Tonic delta/theta power increased over night (classic sleep pressure). Event-related processing weakened: P1 amplitude decreased (latency unchanged), N1 largely unchanged. Delta/theta PLI decreased (reduced trial-to-trial phase consistency), alpha PLI smaller/more limited decreases.	KSS sleepiness increased; RTs slowed, variability/lapses increased.	Visual PVT; tonic EEG power (delta/theta/alpha), occipital ERPs P1/N1, and phase-locking index (PLI)	24 h overnight wakefulness; 8 hourly PVT + EEG sessions (23:30–06:30)	20 healthy young adults (19 EEG analysed; some session-specific N variation)	[89]
No recovery condition; ITPC proposed as sensitive SD biomarker	ITPC markedly reduced across almost entire ssVEP interval under SD (large effects); evoked amplitude lower in some windows; increased ongoing delta/theta/alpha power	More PVT lapses and sleepiness with SD; lower PLV correlated with more PVT lapses and higher theta	Visual ssVEP (7.5 Hz flicker), ITPC/PLV at O1/Oz/O2	Within-subject, counterbalanced; normal sleep vs. 24 h TSD	18 healthy adults (final EEG *n* = 17), 23–32 y	[90]
No recovery; connectivity findings suggest network reorganisation under fatigue	P50 suppression reduced (S1–S2 difference smaller); S1 unchanged, S2 trend larger; altered directed connectivity (↓ occipital→temporal/parahippocampal; ↑ precuneus high-frequency outflow during task)	PVT RT slower, errors ↑; gating change correlated with PVT slowing (r≈0.62)	Auditory paired-click P50 suppression + eLORETA/iCoh effective connectivity	Within-subject RW vs. 36 h TSD	36 healthy men (ERP/connectivity after exclusions)	[91]
Recovery night: CNV remained depressed (incomplete recovery), unlike partial shifts in some other indices	CNV area strongly decreased over deprivation (min around second morning), some subjects showed CPV; CNV stayed low even after recovery night	Subjective fatigue + sleepiness rose sharply and correlated with CNV (symptoms r = −0.79; SSS r = −0.63); CFF/temp weaker correlations	CNV (warning–imperative task at Cz), plus CFF, temp, HR/BP, fatigue scales, SSS	Continuous 36 h TSD, repeated measures every 3 h + post-recovery-night checks	5 healthy male students (selected for high CNV)	[92]
No recovery; suggests impaired control/resource allocation despite limited RT slowing	N1 typical switch effect only; P2 latency delayed in SD; N2 latency delayed and switch-specific amplitude reduction in SD; P3 switch-related reduction present in SD (not control)	Accuracy ↓ with SD; RT and RT switch cost not significantly changed	Task-switching ERPs: N1, P2, N2, P3, LNC	Between-subjects: normal sleep vs. ~24 h TSD	72 students randomised; final *n* = 32 SD, 34 control	[93]
Key modifier = SWS rebound on recovery night intensified/prolonged sleep inertia-like AEP attenuation	Awakening reduced N1–P2 vs. pre-sleep; on recovery night reduction persisted across all awakenings; Night × Site topography shift (↑Fz, ↓Pz/Oz in recovery); N1 latency prolonged after nocturnal awakenings	Simple RT task during AEP; emphasis on hypoarousal after waking	Auditory AEP N1–P2 after awakenings from stage 2	Baseline-with-awakenings vs. recovery night after 2 nights selective SWS suppression	10 healthy men	[94]
Habitual sleep quality moderated subjective changes (good sleepers more affected by acute SD)	In rested group, distraction/reappraisal reduced LPP; in SD group they failed to reduce LPP; suppression ineffective in both	Subjective ratings still showed perceived regulation benefits; SD effect clearer neurally than subjectively	Emotion regulation task (distraction, reappraisal, suppression), post-instruction LPP	Randomised between-subjects; all-night SD vs. normal sleep	51 young adults final: 26 SD, 25 rested	[95]
No recovery; manipulation checks: SSS↑, PVT slowing	N2pc less negative after TSD (weaker orienting); P3 increased (compensatory interpretation); N1 more negative (likely practice effect)	Accuracy ↓, RT variability ↑, mean RT ns; ΔN2pc correlated with Δaccuracy	Visual search ERPs: N1, N2pc, P3	Within-subject baseline vs. 36 h TSD	24 healthy men (behaviour *n* = 23)	[96]
No recovery; combined EMG + ERP dissociates proactive and reactive monitoring deficits	Ne/ERN on full errors reduced in SD; Ne-like on partial errors reduced; reduced differentiation full vs. partial errors (esp. incongruent)	RT ↑, larger Simon cost, more incongruent errors/incorrect activations; proactive suppression weakened in RT distribution/CIAF analyses	Simon task + EMG + response-locked Ne/ERN	Within-subject control vs. 26 h SD, counterbalanced	12 healthy young adults	[97]
8 h recovery sleep partially normalised behaviour and P2 amplitude, but P2 latency remained altered	N1 unchanged; P2 amplitude elevated after TSD, then decreased after 8 h RS toward baseline; P2 latency shortened after TSD and remained short after RS	In SD group: accuracy and efficiency ↓ after TSD, improved after RS; RT ns	Visual 2-back; frontal N1, P2	NS control repeated after normal night; SD group baseline → 36 h TSD → 8 h recovery sleep	40 men randomised (NS *n* = 19, SD *n* = 20 analysed)	[98]
No recovery; shows cortical + brainstem slowing after TSD	P300 latency prolonged, MMN latency prolonged; amplitudes unchanged; BAEP Wave I (bilateral) and Wave V (right) latencies ↑	Selective behavioural deficits (speech-in-noise left ear, music discrimination right ear); correlations: longer P300 latency linked to poorer auditory performance	Auditory CAP tests + P300, MMN, BAEP	Within-subject baseline vs. ~31 h TSD	22 healthy adults	[99]
No recovery; interpreted as compensatory recruitment at later stage	N2 more negative after TSD; P3 amplitude increased after TSD (especially incongruent); P3 latency not changed by sleep	RT shortened (speed-up), accuracy dropped mainly on incongruent trials (speed–accuracy trade-off)	Two-back pronunciation WM; N2 (Fz), P3 (CPz)	Within-subject baseline vs. 36 h TSD	22 healthy men (ERP on subset after artifact exclusions)	[100]
In SD only, theta2 positively correlated with pain/unpleasantness ratings	N2 and N340 amplitudes reduced after SD (esp. painful stimuli); LPP unchanged; theta2 (5–7 Hz, 200–500 ms) reduced	Others’ pain ratings slightly lower after SD; self-unpleasantness largely unchanged; PVT/SSS confirmed sleepiness	Pain-empathy pictures; ERPs N2, N340, LPP + theta TF at CPz	Within-subject crossover normal sleep vs. 24 h TSD	25 healthy students	[101]
No recovery; supports selective impairment of earlier motor-preparation stage	s-LRP compatibility-related amplitude difference disappeared after TSD (early sensory-integration substage impaired); s-LRP onset ns; r-LRP largely preserved (no sleep effect)	Accuracy ↓, RT variability ↑; mean RT ns; compatibility effects intact behaviourally	Stimulus-response compatibility visual search; s-LRP, r-LRP	Within-subject baseline vs. 36 h TSD	24 healthy men (ERP *n* ≈ 23)	[102]
Extra 6 h wakefulness amplified ERP/network deficits; no recovery	Both groups: P200 ↓, P300 latency ↑; 30 h group showed larger N200 increase (more negative) and stronger P300 amplitude reduction vs. 24 h; connectivity: ↓ right insula→left ACC (and →precuneus mismatch), ↑ frontal VLPFC→DLPFC (compensatory) in 30 h	Mismatch accuracy and RT impaired more after 30 h than 24 h	Verbal 2-back; ERPs P200/N200/P300 + eLORETA/iCoh	Between groups; baseline RW then 24 h or 30 h TSD	70 enrolled healthy men; final *n* = 30 (24 h), 34 (30 h)	[103]
Attention condition strongly moderated LEP and habituation effects (distraction abolished P2 habituation difference)	P2 amplitude reduced after TSD in focus/neutral (not distraction); P2 habituation increased after TSD (focus and neutral), faster early decline; N1/N2 less affected	Sleepiness ↑ markedly; pain intensity and unpleasantness ↑ (~40%) despite reduced P2 amplitude	Laser-evoked potentials (LEPs), focus on vertex N2–P2/P2 habituation across blocks	Within-subject crossover habitual sleep vs. one-night TSD	14 enrolled, EEG *n* = 12 healthy students	[104]
Drug modifier: modafinil strongest ERP protection, caffeine partial	Placebo: N2 more negative, P3 amplitude ↓ after TSD; caffeine: P3 amplitude ↓ and N2 latency ↑; Modafinil preserved P3 amplitude, shortened P3 latency after TSD, N2 latency shorter than placebo/caffeine post-TSD	Accuracy dropped after TSD only under placebo; caffeine/modafinil preserved accuracy; RT overall faster under stimulants vs. placebo	Pronunciation 2-back; frontal N2, P3	Randomised double-blind crossover; each session baseline then 36 h TSD + placebo/caffeine/modafinil	16 healthy men	[105]
Key modifier = alcohol dose; mixed synergistic vs. antagonistic interactions depending on measure	CNV: only marginal dose effect, no SD or interaction; EP latencies (esp P200, N330/P450) lengthened with SD and alcohol, with synergistic late-latency increases in SD + moderate alcohol	Categorisation errors showed synergy (largest in SD + moderate alcohol); RT showed antagonistic speeding in combined condition; subjective alertness/anxiety showed antagonistic interactions	Categorisation task with warning tone; CNV + S1 EP components (N130/P200/N330/P450), HR, subjective	Sleep condition within subject (normal vs. 26 h SD); alcohol dose between subject (0, low, moderate)	24 healthy men (8 per alcohol dose)	[106]
No recovery; TSD reduced vigilance/preparatory capacity rather than inducing PINV-like disturbance	No PINV elicitation after TSD; CNV amplitude decreased; AEP latencies P1/N1 ↑; N1 and P2 amplitudes ↓	RT slowed; HR decrease ns	CNV/PINV paradigm + auditory EPs to S1	Within-subject pre vs. 48 h TSD	19 healthy men (subsets for some analyses)	[107]
Key modifier = future performance grouping (HIGH/NOR/LOW); P3/delta-mediated ΔRT→performance link	Incongruent Stroop P3 at C3 linked to SD-induced RT change and future performance; delta band (1–4 Hz) power within P3 window differed by performance group	SD raised anxiety/cortisol to competition-like levels; Δincongruent Stroop RT under SD predicted later competition outcome	Stroop + EEG (P3, TF delta), plus stress (anxiety/cortisol)	24 h SD used as competition-like stressor; baseline and SD EEG; later real competition follow-up	65 athletes (35 college test set + 30 pro-validation set)	[108]
Important modifier = upcoming REM vs. no-REM nap in narcoleptics (AER pattern differs)	In normals, SD increased N1–P2 and P2–N2 amplitudes; narcolepsy split by upcoming nap REM: no-REM naps showed larger N1–P2 (SD-like), REM naps showed reduced amplitudes	Focus was physiological sleepiness; naps classified by MSLT/REM occurrence	Long-latency auditory evoked responses N1–P2, P2–N2 before naps	Controls tested rested and after full-night SD; narcoleptics repeatedly across MSLT naps	15 narcoleptics + 10 controls	[109]
No nap/recovery; combined EEG + behaviour improved individual detection of impairment	N170 amplitude ↓ and latency ↑, P215 ↓, LPC amplitude ↓ by ~1:30 AM; later slow wave unchanged; tonic workload theta/alpha effects preserved	Sleepiness ↑ (Karolinska), d′ ↓, RT ↑, RT variability ↑; decrements emerged soon after usual bedtime	Spatial n-back; task ERPs N170, P215, LPC, resting EEG, workload spectra	Within-subject repeated overnight wakefulness (~15–21 h awake) vs. daytime baseline	16 healthy young adults	[110]
Recovery night did not erase prior SD disadvantage; PSG SWS showed paradoxical/celling-related correlations	Sleep group showed N400 attenuation (less negative) for old-intact pairs from pre→post (stronger semantic links); Wake group no reliable N400 attenuation; Wake group post-test N400 more negative	Sleep group improved recognition accuracy/RT; Wake group OI accuracy worsened/no RT gains; subjective sleepiness ns at tests	Word-pair associative recognition; N400 (target-locked), plus N1/P2	Learn at night; Sleep group slept, Wake group overnight SD; both had recovery night before post-test	30 students (Sleep *n* = 15, Wake-deprived *n* = 15)	[111]
Drug modifier = caffeine; stronger on early processing/inhibition-related N2 than on P3	Caffeine mainly enhanced early components after TSD (Go/No-Go P2 amplitude ↑; No-Go N2 amplitude ↑ and N2 latency shortened at Fz); P3 mostly showed TSD effects, limited caffeine normalisation	Go hit rate better with caffeine than placebo after TSD; RT effects mixed; No-Go FA ns	Go/No-Go ERPs: P2, N2, P3	Double-blind within-subject crossover; baseline then 36 h TSD with caffeine vs. placebo	16 healthy men	[112]
Short nap produced partial ERP recovery without behavioural recovery	TSD: N2 latency prolonged, P3 latency prolonged, P3 amplitude increased (compensatory); After 1 h nap: P3 latency shortened toward baseline, N2 amplitude increased (early neural rebound), amplitudes otherwise mixed	TSD worsened Go RT, Go hit rate, No-Go FA; 1 h nap did not significantly restore behaviour	Go/No-Go ERPs N2, P3 (frontal/frontocentral)	Within-subject: baseline → 30 h TSD → 1 h recovery nap	27 recruited healthy men; final *n* = 22	[113]
Practice/automaticity (CM vs. VM) was key modifier: wakefulness harmed global efficiency more than core search slopes	P300 latency increased and P300 amplitude decreased across wakefulness; effects tracked task difficulty (VM, set size, target absence)	RT ↑, accuracy ↓ (esp. VM/high load), nonresponses/lapses ↑; but search slopes (automatic vs. controlled process markers) largely preserved	CM vs. VM memory/visual search; P300	Intensive training then overnight extended wakefulness (multiple sessions)	10 right-handed men	[114]
Athlete sample; network reorganisation suggests partial compensation but insufficient	P3 amplitude decreased after TSD; right-hemisphere P3 > left (amplitude/latency effects); β-band connectivity: many decreases (esp. frontal–occipital) plus fewer increases (compensatory)	RT ↑, correct responses/sec ↓, accuracy ns	Spatial 2-back + frontal P3 and β-band PLV connectivity	Within-subject baseline after 8 h sleep vs. 36 h TSD	20 male table tennis athletes	[115]

**Table 2:** ROBINS-I risk of bias assessment.

Bias in Selection of the Reported Result	Bias in Measurement of Outcomes	Bias Due to Missing Data	Bias Due to Deviations from Intended Interventions	Bias in Classification of Interventions	Bias in Selection of Participants into the Study	Bias Due to Confounding	Study
Moderate	Moderate	Low	Moderate	Low	Moderate	Serious	[34]
Moderate	Moderate	Low to Moderate	Moderate	Low	Moderate	Serious to Critical	[37]
Serious	Serious	Serious	Moderate to Serious	Moderate	Moderate	Critical	[38]
Moderate	Moderate	Low	Moderate	Low	Moderate	Serious	[39]
Moderate	Low to Moderate	Moderate	Moderate	Low	Low to Moderate	Moderate to Serious	[41]
Moderate	Moderate	Moderate	Low to Moderate	Low	Moderate to Serious	Serious	[42]
Moderate to Serious	Moderate	Serious	Moderate	Low	Moderate	Serious	[43]
Serious	Moderate	Moderate	Moderate	Low	Moderate	Serious	[44]
Some concerns to Moderate	Moderate	Moderate	Moderate	Low	Moderate	Serious	[46]
Moderate	Moderate	Moderate	Moderate	Low	Moderate	Serious	[47]
Serious	Moderate	Moderate	Moderate	Low	Moderate	Serious	[48]
Moderate to Serious	Moderate	Moderate	Moderate	Low	Moderate	Serious	[49]
Moderate	Moderate	Serious	Moderate	Low to Moderate	Moderate	Serious	[50]
Serious	Moderate	Moderate	Moderate	Low to Moderate	Moderate	Serious	[53]
Moderate to Serious	Moderate	Moderate to Serious	Moderate	Low	Moderate	Serious	[55]
Serious	Moderate	Serious	Moderate	Moderate	Moderate	Critical	[56]
Moderate	Moderate	Serious	Low to Moderate	Low	Moderate	Serious	[60]
Serious	Low to Moderate	Low	Moderate	Low	Moderate	Critical	[61]
Moderate	Moderate	Low	Moderate to Serious	Low	Moderate	Serious	[62]
	Subjective outcomes (SSS, ESS): Serious	Moderate	Serious	Low	Moderate	Serious	[63]
	ERP/CNV latencies/amplitudes: Moderate						
Moderate to Serious	Moderate	Low	Moderate	Moderate	Moderate	Moderate to Serious	[64]
Moderate	Low	Moderate to Serious	Low to Moderate	Low	Low	Moderate	[65]
Moderate to Serious	Moderate	Moderate	Moderate	Low	Moderate	Serious	[66]
Moderate to Serious	Moderate	Moderate	Moderate	Low	Moderate	Serious	[67]
Serious	Moderate	Low for behavioural/ERP; Moderate for FC	Low to Moderate	Low	Moderate	Serious	[69]
Serious	Moderate	Serious	Moderate	Low	Moderate	Critical	[71]
Moderate	Low (ERP)/Moderate (self-report outcomes)	Low to Moderate	Moderate	Low	Moderate	Serious	[74]
Moderate	Moderate	Low to Moderate	Moderate	Low to Moderate	Moderate	Serious	[77]
Moderate to Serious	Low to Moderate	Moderate	Moderate	Low	Low to Moderate	Moderate	[81]
Serious	Moderate	Low to Moderate	Moderate	Low	Moderate to Serious	Serious to Critical	[83]
Moderate	Low	Low to Moderate	Moderate	Low	Low	Moderate	[84]
Moderate	Moderate	Serious	Moderate	Low	Moderate	Serious	[85]
Moderate	Moderate	Serious	Moderate	Low to Moderate	Moderate	Moderate to Serious	[86]
	Behavioural outcomes: Low to Moderate	TSD: Moderate	Both: Low to Moderate	Both: Low	Both: Low to Moderate	TSD (Experiment 1): Serious SR (Experiment 2): Moderate	[87]
	EEG outcomes: Moderate	SR: Moderate to Serious (EEG outcomes)					
Moderate	Moderate	Moderate	Moderate	Low	Moderate	Serious	[88]
Serious	Moderate	Moderate	Low to Moderate	Low	Low to Moderate	Serious	[89]
Moderate	Moderate	Moderate to Serious	Moderate	Low to Moderate	Moderate	Serious	[91]
Serious	Moderate	Moderate	Moderate	Low	Serious	Serious	[92]
Serious	Moderate	Low to Moderate	Moderate	Low	Moderate	Serious	[94]
Moderate to Serious	Moderate	Moderate	Moderate	Low to Moderate	Moderate	Serious	[96]
Moderate	Moderate	Low to Moderate	Moderate	Low	Low	Moderate	[97]
Serious	Moderate	Low	Moderate	Low	Moderate	Serious	[98]
Moderate to Serious	Moderate	Low	Moderate	Moderate	Moderate	Serious	[99]
Moderate	Moderate	Moderate	Moderate	Low	Moderate	Serious	[100]
Serious	Moderate	Moderate	Moderate	Low	Low to Moderate	Moderate to Serious	[101]
Moderate to Serious	Moderate	Moderate	Moderate	Low	Moderate	Serious	[102]
Serious	Moderate	Moderate	Moderate	Low	Moderate to Serious	Serious	[103]
Moderate	Moderate (Serious for subjective pain ratings)	Moderate	Moderate	Low	Low to Moderate	Moderate	[104]
Moderate to Serious	Moderate	Serious	Moderate	Low	Low to Moderate	Moderate to Serious	[106]
Serious	Moderate	Serious	Moderate to Serious	Low	Moderate	Serious	[107]
Serious	Serious	Low for main outcomes, Moderate for EEG analyses	Low to Moderate	Low to Moderate	Moderate to Serious	Serious	[108]
Moderate to Serious	Moderate	Moderate	Moderate	Low to Moderate	Moderate to Serious	Serious	[109]
Moderate	Moderate	Moderate	Moderate	Low	Low to Moderate	Serious	[110]
Moderate	Moderate	Moderate to Serious	Moderate	Low	Moderate	Serious	[113]
Moderate	Moderate	Moderate	Moderate	Low to Moderate	Moderate to Serious	Serious	[114]
Serious	Moderate	Moderate to Serious	Moderate	Low	Serious	Critical	[115]

**Table 3:** Rob 2 risk of bias assessment.

Bias in Selection of the Reported Result	Bias in Measurement of the Outcome	Bias Due to Missing Outcome Data	Bias Due to Deviations from Intended Interventions	Bias Arising from the Randomisation Process	Study
Some concerns	Low risk	Low risk	Low risk	Some concerns	[35]
Some concerns	Low risk to Some concerns	Some concerns	Some concerns	Some concerns	[36]
Some concerns	Some concerns	Low risk	High risk	Some concerns	[40]
Some concerns	Low risk	Low risk	Some concerns	Some concerns	[45]
High risk	Low risk	Some concerns	Some concerns	Some concerns	[51]
High risk	Some concerns	Low risk	Some concerns	Some concerns	[52]
Some concerns	Some concerns	High risk	Some concerns	Some concerns	[54]
Some concerns	EEG/ERP outcomes: Low risk Behavioural task outcomes: Some concerns	Low risk	Low risk to Some concerns	Some concerns	[57]
Some concerns	Some concerns	Some concerns	Some concerns	Some concerns	[58]
Some concerns	Low risk	Some concerns	Low risk	Some concerns	[59]
Some concerns	Low risk	High risk	Some concerns	Some concerns	[68]
Some concerns	Some concerns	Some concerns	Some concerns	Some concerns	[70]
Some concerns	Some concerns	Behavioural outcomes (accuracy/RT): Some concerns P300 ERP outcome: High risk/Some concerns leaning high (depending on strictness)	Some concerns	Some concerns	[72]
Some concerns	Behavioural/ERP outcomes: Low risk to Some concerns Subjective outcomes (effort/confidence): High risk	Behavioural outcomes: Low risk ERP outcomes: Some concerns	High risk	Some concerns	[73]
Some concerns	Low risk (ERP outcomes), Some concerns (behavioural outcomes)	Some concerns	High risk	Some concerns	[75]
High risk	Some concerns	Some concerns	Some concerns	Some concerns	[76]
Some concerns	Low risk	High risk	Some concerns	Some concerns	[78]
High risk	Some concerns	Some concerns	High risk	Some concerns	[79]
Some concerns	Some concerns	High risk	Some concerns	Some concerns	[80]
High risk	Behavioural outcomes: Low risk to Some concerns ERP outcomes: Some concerns	Behavioural outcomes: Some concerns ERP outcomes: High risk	Some concerns	Some concerns	[82]
Some concerns	Some concerns	Low risk	Some concerns	Some concerns	[90]
Behavioural outcomes: Some concerns EEG/ERP/time-frequency outcomes: High risk	Behavioural outcomes (accuracy, RT): Low risk EEG/ERP/time-frequency outcomes: Some concerns to High	Some concerns	Some concerns	Some concerns	[93]
Some concerns	ERP/LPP outcome: Low risk Valence/arousal self-report outcome: Some concerns	Some concerns	Some concerns (for subjective outcomes), Low risk to Some concerns (for ERP)	Some concerns	[95]
Some concerns	Low risk	Some concerns	Low risk	Some concerns	[105]
Some concerns	Behavioural outcomes (recognition accuracy, RT): Some concerns ERP outcomes (N400): Some concerns	Low risk	Some concerns	Some concerns	[111]
High risk	Low risk	Low risk	Some concerns	Some concerns	[112]

**Table 4:** Strength and interpretation of evidence by ERP domain.

ERP Domain	Direction of Evidence	Synthetic Interpretation	Strength of Conclusion
P300/P3	Frequently delayed and/or reduced; occasional compensatory increases	Most consistent marker of impaired late stimulus evaluation, attentional allocation, and context updating	Strong
P1/N1/MMN/P50	Often preserved, but sometimes delayed, weakened, or less phase-locked	Early sensory/pre-attentive processing is relatively resilient but not immune	Moderate
P2/N2	Task-dependent amplitude and latency changes	Sleep loss affects intermediate attentional selection, conflict detection, and working memory updating	Moderate
CNV	Often reduced or delayed	Impaired preparatory attention and expectancy under sustained wakefulness	Moderate
ERN/Ne/Pe	Often reduced, especially Pe and sometimes ERN/Ne	Weakened error monitoring, conscious error evaluation, and adaptive control	Moderate
LPP/N400/memory-related components	Variable and task-specific	Emotional and memory processing are affected, but conclusions depend strongly on paradigm	Limited to moderate
Recovery/countermeasures	Partial and component-specific normalisation	Behavioural recovery may exceed neural recovery; amplitude and latency recover differently	Moderate

## Data Availability

No new data were created or analysed in this study. Data sharing is not applicable to this article.

## Supplementary Materials

The following supporting information can be downloaded at: https://www.mdpi.com/article/10.3390/jcm15124576/s1, Table S1: PRISMA 2020 checklist [271].
